# Electron transport phosphorylation in rumen butyrivibrios: unprecedented ATP yield for glucose fermentation to butyrate

**DOI:** 10.3389/fmicb.2015.00622

**Published:** 2015-06-24

**Authors:** Timothy J. Hackmann, Jeffrey L. Firkins

**Affiliations:** ^1^Department of Animal Sciences, University of Florida, Gainesville, FLUSA; ^2^Department of Animal Sciences, The Ohio State University, Columbus, OHUSA

**Keywords:** rumen microbiology, butyrivibrios, electron transport phosphorylation, ATP yield, energy conservation

## Abstract

From a genomic analysis of rumen butyrivibrios (*Butyrivibrio* and *Pseudobutyrivibrio* sp.), we have re-evaluated the contribution of electron transport phosphorylation (ETP) to ATP formation in this group. This group is unique in that most (76%) genomes were predicted to possess genes for both Ech and Rnf transmembrane ion pumps. These pumps act in concert with the NifJ and Bcd-Etf to form a electrochemical potential (ΔμH^+^ and ΔμNa^+^), which drives ATP synthesis by ETP. Of the 62 total butyrivibrio genomes currently available from the Hungate 1000 project, all 62 were predicted to possess NifJ, which reduces oxidized ferredoxin (Fd_ox_) during pyruvate conversion to acetyl-CoA. All 62 possessed all subunits of Bcd-Etf, which reduces Fd_ox_ and oxidizes reduced NAD during crotonyl-CoA reduction. Additionally, 61 genomes possessed all subunits of the Rnf, which generates ΔμH^+^ or ΔμNa^+^ from oxidation of reduced Fd (Fd_red_) and reduction of oxidized NAD. Further, 47 genomes possessed all six subunits of the Ech, which generates ΔμH^+^ from oxidation of Fd_red_. For glucose fermentation to butyrate and H_2_, the electrochemical potential established should drive synthesis of ∼1.5 ATP by the F_0_F_1_-ATP synthase (possessed by all 62 genomes). The total yield is ∼4.5 ATP/glucose after accounting for three ATP formed by classic substrate-level phosphorylation, and it is one the highest yields for any glucose fermentation. The yield was the same when unsaturated fatty acid bonds, not H^+^, served as the electron acceptor (as during biohydrogenation). Possession of both Ech and Rnf had been previously documented in only a few sulfate-reducers, was rare in other rumen prokaryotic genomes in our analysis, and may confer an energetic advantage to rumen butyrivibrios. This unique energy conservation system might enhance the butyrivibrios’ ability to overcome growth inhibition by unsaturated fatty acids, as postulated herein.

## Introduction

In anaerobic prokaryotes, SLP was long-thought to be the primary mechanism for ATP synthesis and energy conservation. ETP, in contrast, was thought to be minor. It was thought to be coupled only to dissimilatory reduction of inorganic elements or compounds (e.g., sulfate), fumarate reduction, organic acid decarboxylation, eﬄux of organic acid end-products, and methanogenesis (Gottschalk, 1986; White, 2007). Evidence supported that ETP was coupled to reductive acetogenesis, also, but the full mechanism could not be elucidated (Müller, 2003).

Recently, mechanisms of ETP have been elucidated in more pathways of anaerobic prokaryotes. These pathways include (1) acetate formation during glucose fermentation in *Pyrococcus furiosus* (Sapra et al., 2003); (2) caffeyl-CoA reduction and H_2_ oxidation during caffeate respiration in *Acetobacterium woodi* (Imkamp et al., 2007; Bertsch et al., 2013); (3) crotonyl-CoA reduction during butyrate formation in *Clostridium* sp. and *Acidaminococcus fermentans* (Herrmann et al., 2008; Li et al., 2008); (4) H_2_ oxidation during reductive acetogenesis in *A. woodi, Clostridium ljungdahlii*, and *Moorella thermoacetica* (Schuchmann and Müller, 2014); and (5) CO oxidation during formation of acetate and other end products in *C. autoethanogenum* (Wang et al., 2013a).

In these pathways, ETP involves the transmembrane ion pumps Ech or Rnf. These pumps generate a transmembrane electrochemical potential (ΔμH^+^ or ΔμNa^+^) from redox cofactors generated by pathway enzymes. Specifically, Ech is a hydrogenase that generates ΔμH^+^ from oxidation of Fd_red_ (Welte et al., 2010). Rnf generates ΔμH^+^ or ΔμNa^+^ from simultaneous oxidation of Fd_red_ and reduction of NAD_ox_; (Biegel and Müller, 2010; Schlegel et al., 2012; Tremblay et al., 2013), in a mechanism known as flavin-based electron bifurcation (Buckel and Thauer, 2013). Redox cofactors are generated by pathway enzymes using conventional mechanisms and enzymes recently discovered to generate redox cofactors by the mechanism of electron bifurcation (Buckel and Thauer, 2013). The electrochemical potential established by Ech and Rnf in turn drives F_0_F_1_-ATP synthase to form ATP by ETP (Welte et al., 2010; Buckel and Thauer, 2013), or, alternatively, it could drive solute transport or motility. Organisms appear to possess either Ech or Rnf, and reports of organisms possessing both are rare (Pereira et al., 2011; Weghoff et al., 2015).

While searching rumen prokaryotic genomes for genes supporting ETP, we found that the rumen butyrivibrios [*Butyrivibrio* and *Pseudobutyrivibrio* sp. (Cotta and Forster, 2006)] uniquely possessed genes for both Ech and Rnf. We suggest Ech and Rnf function in concert with the NifJ protein and Bcd–Etf complex [pathway enzymes generating redox cofactors; (Buckel and Thauer, 2013; Gutekunst et al., 2014)], permitting unprecedented ATP yield during glucose fermentation to butyrate. Activity of Ech and Rnf, if experimentally confirmed, could give members of this group an energetic advantage and help explain their metabolic flexibility.

The butyrivibrios are the best characterized group of bacteria that biohydrogenate LA to VA; *Butyrivibrio proteoclasticus* represents the only known clade that biohydrogenates VA fully to SA through membrane-associated reductases (Jenkins et al., 2008). A hypothesis will be justified that *de novo* synthesis of long-chain saturated fatty acids increases to counteract this fluidization of the outer membrane. Because electron transfer mechanisms reoxidize all NAD_red_ from glucose fermentation to butyrate, though, *de novo* synthesis of fatty acids would be in competition with butyrate production for reducing equivalents, particularly as proposed for transhydrogenation mechanisms generating NADP_red_ from NAD_red_. This theory attempts to explain why abrupt dosing of LA did not compromise membrane integrity but extended lag time for growth while coenzyme A-esterified intermediates in the butyrate production pathway decreased prior to ATP depletion in a strain of *B. fibrisolvens* (Maia et al., 2010).

## Materials and Methods

We analyzed all 62 genomes of *Butyrivibrio* and *Pseudobutyri vibrio* sp. sequenced in the Hungate 1000 project (Creevey et al., 2014) and available in the IMG database (Markowitz et al., 2014). We identified proteins involved in butyrate fermentation and ETP by searching for KO (Kanehisa et al., 2014), COG (Galperin et al., 2015), and pfam protein families (Finn et al., 2014) IDs as indicated parenthetically below. Specifically, we searched for ATPF0ABC and ATPF1ABDEG (K02108 to K02115); Bcd-EtfAB (K00248, K03521, and K03522); EchABCDEF (K14086 to K14091); EhaA-R (K14092 to K14109); EhbA-P (K14110 to K14124); Eno (K01689); the Hyd proteins HydA (K00532), HydA large subunit (K00533), HydB (K00534), HydC (K06441), and HydA1B1G1 (K17997 to K17999); the Fdh proteins FDH (K00122), FdhF (K00123), FdhB (K00125), and FdsD (K00126); the glyoxalase proteins GloA (K01759) and GloB (COG0491); the lactate dehydrogenases LdhA (K03778) and Ldh (K00016); MbhLKJ (K18016, K18017, K18023); MsgA (K01734); MvhADG/HdrABC (K14126 to K14128 and K03388 to K03390); NifJ (K03737); PflD (K00656); PorABDG (K00169 to K00172); and RnfABCDEG (K03612 to K03615, K03617, and COG2878). FdhF was searched as both the Fdh alone and as a complex with (1) Hyl (FdhF-HylABC; K00123, pfam10588, K00334, K00335) or (2) Hyc (FdhF-HycBCDEFG; K00123 and K15827 to K15832). We searched acetyl-CoA carboxylase (AccABCD; K01961, K02160, K01962, K01963), FabD (K00645), FabF (K09458), FabG (K00059), FabH (K00648), FabK (K02371), FabZ (K02372), and acyl transferases (glycerol-3-phosphate acyltransferases, PlsX, K03261, PlsY, K08591; 1-acyl-sn-glycerol-3-phosphate acyltransferase, PlsC, K00655). We searched TCA cycle intermediates (aconitase, AcnA, K01681; citrate synthase, CS, K01647; NADP-isocitrate dehydrogenase, IDH1, K00031; α-ketoglutarate dehydrogenase complex, korABDG, K00174-K00177; malate dehydrogenase, Mdh, K00024; malic enzyme, MaeA, K00027; and succinate dehydrogenase, sdhAB, K00239, K00240) and transhydrogenase reactions (soluble transhydrogenase, SthA, K00322; pyrimidine nucleotide transhydrogenase, PntAB, K00324, K00325; and hydroxyl-ketoacid transyhdrogenase, ADHFE1, K11173; pyruvate kinase, Pyk, K00873; pyruvate-phosphate dikinase, Ppdk, K01006; phosphoenolpyruvate carboxykinase, PckA, K01610; oxaloacetate decarboxylase, OadAB, K01571, K01572; glutamate dehydrogenase, GdhA, K00262; and glutamate synthase, GltD, K00266). Here and throughout, searches were conducted between 4 and 20 May, 2015.

The KO ID was chosen for searches first, and the COG ID was searched only when the KO ID produced few to no search hits, even for genomes known to possess protein activity. *Prevotella bryantii* B_14_ synthesizes methylglyoxal (Russell, 1992), but GloB was missing when searching for the KO ID (K01069). *A. woodi* has Rnf activity (Biegel and Müller, 2010), but RnfB was missing when searching for K03616. These proteins were found in the respective genomes when searching for the COG IDs (listed above). The pfam ID was searched only when a KO ID was not available for the protein (HylA).

For comparison, we searched for all proteins above for both genomes of non-rumen butyrivibrios that were available in IMG database. Additionally, using the IMG database, we searched for Ech and Rnf in (1) 218 genomes of non-butyrivibrio rumen prokaryotes in the Hungate 1000 project, (2) all 47 of *Desulfovibrio* genomes available, (3) all 451 genomes of *Clostridium* sp. available, (4) 28 genomes of short-chain fatty acid degraders previously analyzed by Worm et al. (2014), and (5) 10 of the 20 genomes of lactate fermenters previously analyzed by Weghoff et al. (2015; the authors did not explicitly identify the other 10 genomes).

## Results

Our analysis of rumen butyrivibrio genomes suggests that butyrivibrios possess genes for generating ATP by ETP during glucose fermentation to butyrate. Of 62 total genomes sequenced in the Hungate 1000 project and available on the IMG database, all 62 were predicted to possessed all subunits of the F_0_F_1_-ATP synthase (ATPF0ABC and ATPF1ABDEG), 62 possessed all subunits of Bcd-Etf (Bcd and EtfAB), 62 possessed NifJ, and 61 possessed all subunits of RnfABCDEG. Additionally, 47 genomes possessed all six subunits of EchABCDEF. These same genomes also possessed Rnf, making 47 genomes (76% of total) that possessed both Ech and Rnf.

EchD and EchF were the subunits of Ech missing most often, with each individually absent in seven genomes. Interestingly, EchD was individually absent in all six *B. fibrisolvens* genomes, and only in one other genome (*Butyrivibrio* sp. TB) was it also absent. EchF was absent over a broader range of genomes; it was individually absent in *Pseudobutyrivibrio ruminis* (two genomes), *Pseudobutyrivibrio xylanivorans* (one genome), unclassified *Pseudobutyrivibrio* sp. (one genome), and unclassified *Butyrivibrio* sp. (one genome). In only one other genome was any Ech subunit absent; *Butyrivibrio* sp. NC3005 lacked all subunits.

No genome possessed genes for PorABDG, a Por similar in function to NifJ. No genome possessed genes for EhaA-R, EhbA-P, HydA (K00532), HydB, HydC, HydA1B1G1, MbhLKJ, or MvhADG/HdrABC, which are similar in function to Ech in the respect that they are hydrogenases and Fd-dependent. Twenty genomes possessed the gene for the HydA large subunit (K00533), part of another Fd-dependent hydrogenase, but they did not possess other subunits (HydB, HydC).

**FIGURE 1:**
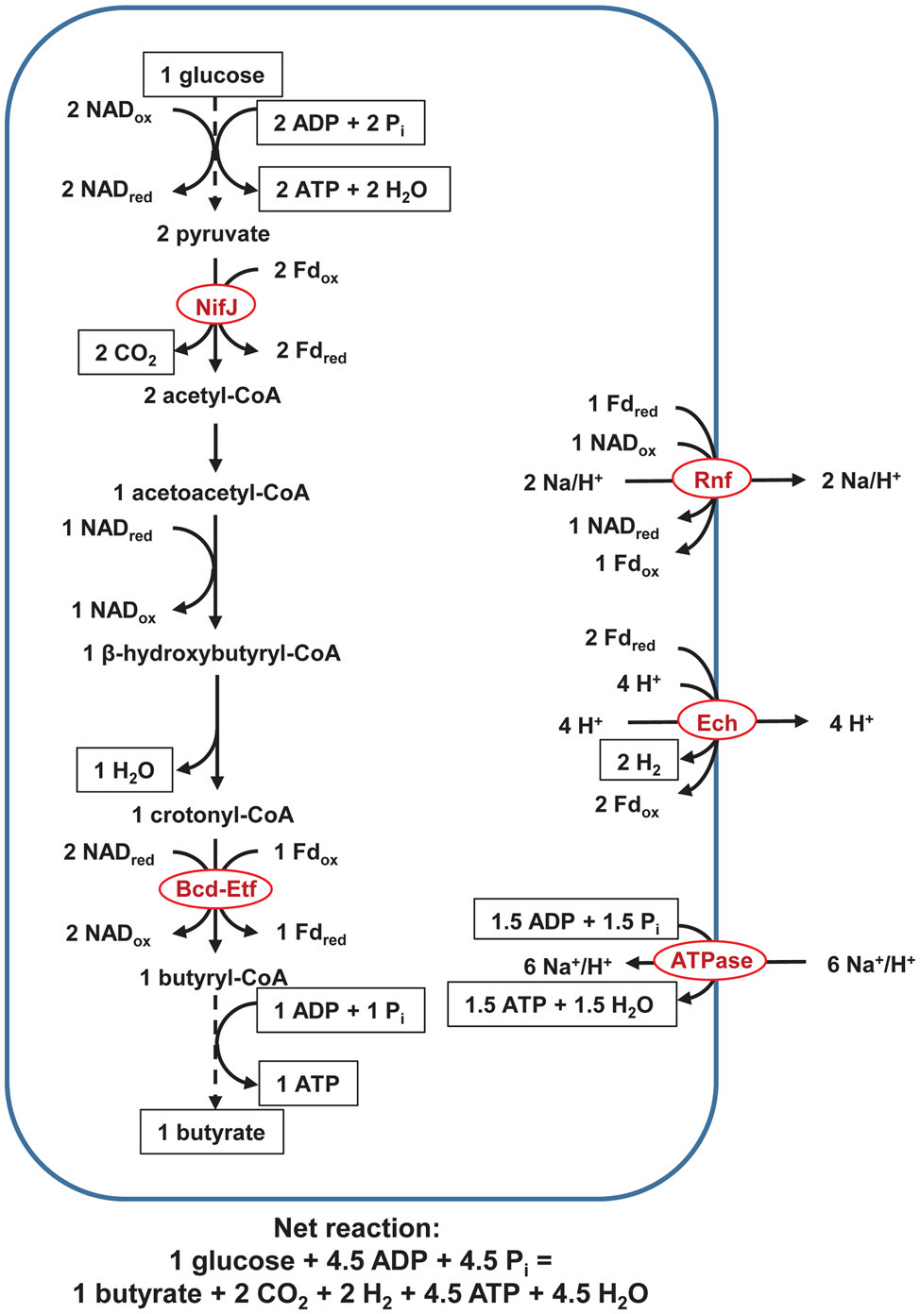
**Pathway of fermentation of glucose to butyrate in rumen butyrivibrios with NifJ and with H^+^ as an electron acceptor.** Three ATP are formed by SLP, as long-recognized, but up to an additional ∼1.5 ATP are formed by ETP. Dashed lines represent steps of the pathway condensed for brevity. In the equation of the net reaction, hydrogen atoms and charges are not balanced, following the convention of Alberty (2003) for representing biochemical reactions. ATPase = F_0_F_1_-ATP synthase. See text for details and additional abbreviations.

**FIGURE 2:**
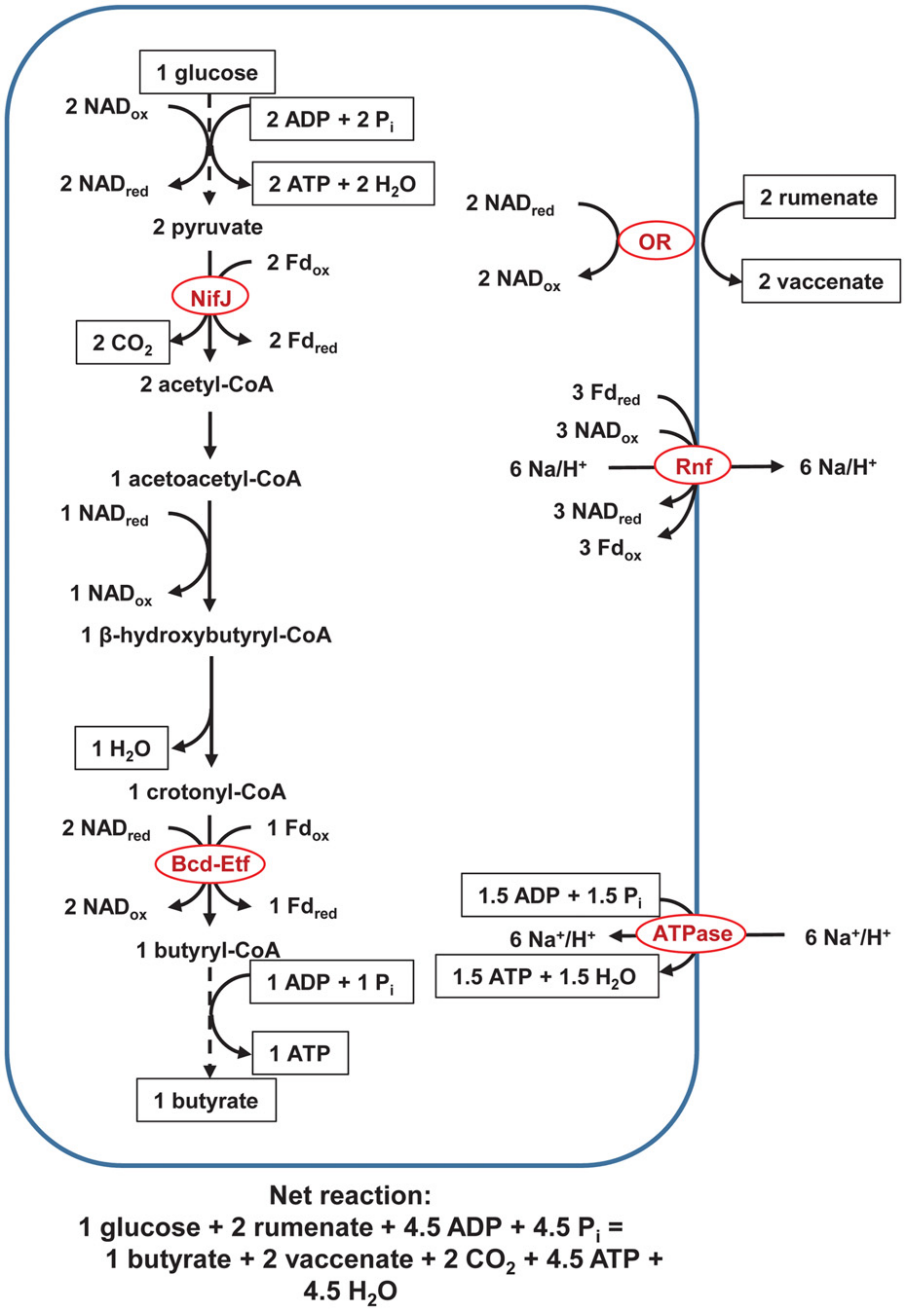
**Pathway of fermentation of glucose to butyrate in rumen butyrivibrios with NifJ and with an unsaturated fatty acid bonds as an electron acceptor.** Pathway is identical to that in **Figure 1**, except reduction of rumenate to vaccenate by an oxidoreductase is additionally shown. ATPase = F_0_F_1_-ATP synthase and OR = fatty acid oxidoreductase. Dashed lines represent steps of the pathway condensed for brevity. See text for details and additional abbreviations.

**FIGURE 3:**
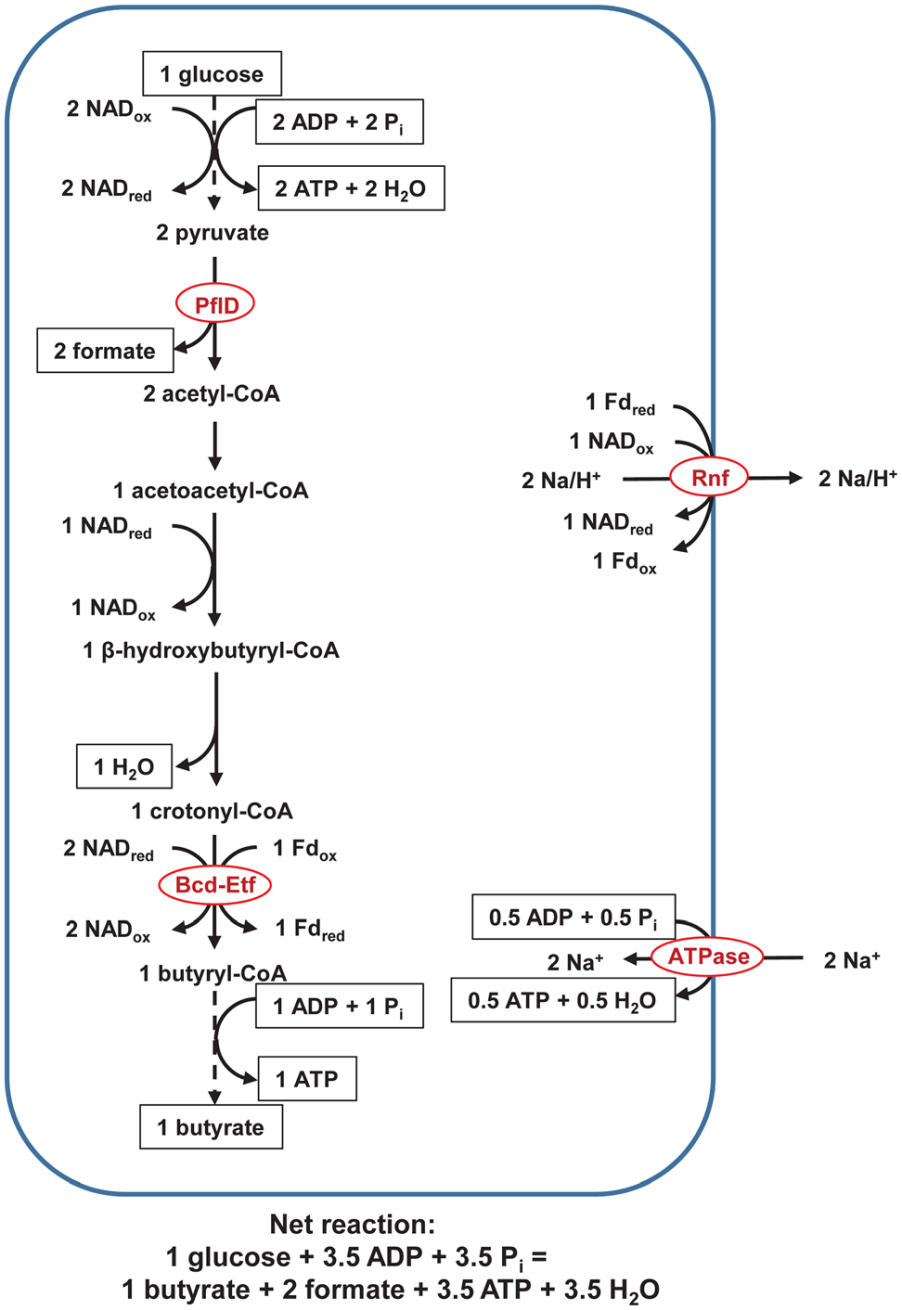
**Pathway of fermentation of glucose to butyrate in rumen butyrivibrios with PflD.** Pathway is identical to that in **Figure 1**, except PflD replaces NifJ. ATPase = F_0_F_1_-ATP. Dashed lines represent steps of the pathway condensed for brevity. See text for details and additional abbreviations.

In our proposed pathway of butyrate and H_2_ from glucose (**Figure 1**), NifJ generates Fd_red_ during pyruvate conversion to acetyl-CoA. Bcd-Etf generates Fd_red_ and NAD_ox_ during crotonyl-CoA reduction to butyryl-CoA. Ech oxidizes Fd_red_, and Rnf oxidizes Fd_red_ while reducing NAD_ox_. In so doing, Ech and Rnf pump H^+^ and Na^+^, form ΔμH^+^ and ΔμNa^+^, and regenerate NAD_red_ and Fd_ox_ to achieve a balanced redox. The electrochemical potential drives ATP synthesis by F_0_F_1_-ATP synthase to yield ∼1.5 ATP/glucose. An additional three ATP is formed by classic SLP. Specifically, two ATP are formed during the EMP pathway, and one ATP is formed either by (1) butyrate kinase or (2) butyryl-CoA/acetate CoA transferase, phosphotransacetylase, and acetate kinase (not shown in **Figures 1–3**). In total, ETP and SLP yield ∼4.5 ATP/glucose.

In the pathway above (**Figure 1**), H^+^ serves as an electron acceptor. We present a second pathway in which an unsaturated fatty acid serves as an electron acceptor instead (**Figure 2**). In this pathway, fatty acid oxidoreductase couples reduction of rumenate to vaccinate, forming NAD_ox_. As a consequence of the extra NAD_ox_ formed, Rnf alone, not Ech, is active; only Rnf can reduce the extra NAD_ox_ and regenerate NAD_red_ to achieve a balanced redox. The ATP yield is not affected, with ETP still yielding ∼1.5 ATP and SLP yielding 3 ATP/glucose.

The pathways with NifJ are not the only one possible, and an alternate pathway involves a pyruvate formate lyase, PflD (**Figure 3**). It was possessed by 61 of the 62 genomes. If the decarboxylation of acetyl-CoA is catalyzed by PflD instead of NifJ, two fewer Fd_red_ are generated, only Rnf is active, and ATP yield decreases from 4.5 to 3.5/glucose (**Figure 3**).

The pathway with PflD (**Figure 3**) depicts formate as an end product, but if a Fdh were present, formate would be oxidized to CO_2_ and H_2_. Five genomes possessed FdhF; no other Fdh (FDH, FdhB, FdsD) was present. Of the five genomes with FdhF, none possessed a full Fdh-hydrogenase complex (FdhF-HylABC or FdhF-HycBCDEFG). Formate would indeed be the predicted the end product for the pathway with PflD.

Pathways presented so far presume possession of a full EMP pathway (c.f., **Figures 1–3**). However, one EMP pathway enzyme (the enolase Eno) was missing in 25 genomes (not shown). The methylglyoxal pathway would be an alternate to a full EMP pathway (Supplementary Figure S1). All 62 genomes possessed MgsA, 45 possessed GloA, and 62 possessed GloB. Another methylglyoxal pathway enzyme, D-lactate dehydrogenase (LdhA), was found only in one genome, but it could be substituted by (1) L-lactate dehydrogenase (Ldh) and (2) a lactate racemase (Supplementary Figure S1). Ldh was found in 51 genomes, but possession of a racemase could not be confirmed because it is not in KEGG, COG, pfam or other databases. Presuming that it is complete, the methylglyoxal pathway would yield four fewer ATP than does the full EMP pathway (c.f., Supplementary Figure S1). Consequently, if the methylglyoxal pathway were active, the pathway of butyrate and H_2_ from glucose would yield either 0.5 ATP/glucose (with NifJ) or -0.5 ATP/glucose (with PflD).

Outside the butyrivibrios, few rumen prokaryotes possessed both Ech and Rnf. Of the 218 non-butyrivibrio genomes sequenced in the Hungate 1000 project, 134 possessed all subunits of Rnf and 16 possessed all subunits of Ech (c.f., Supplementary Table S1). Of those, 10 possessed both Rnf and Ech. These genomes belonged exclusively to strains of *Desulfovibrio desulfuricans* (one strain) and unclassified Lachnospiraceae (nine strains).

Only two non-rumen butyrivibrio genomes were in the IMG database, and they were sequenced in the Human Microbiome Project. Both genomes (*B. crossotus* DSM 2876, *B. fibrisolvens* 16/4) had all subunits of Bcd-Etf, NifJ, and PflD. One genome (*B. crossotus* DSM 2876) had all subunits of F_0_F_1_-ATP synthase and all subunits of Rnf. Neither genome had all subunits of Ech.

Of 47 genomes of *Desulfovibrio* sp. available in the IMG database, 17 genomes possessed all subunits of Rnf. Fifteen genomes possessed all subunits of Ech, but only five of those genomes (*D. cuneatus* DSM 11391, *D. desulfuricans desulfuricans* ATCC 27774, *D. desulfuricans* DSM 7057, *Desulfovibrio* sp. 3_1_syn3, *Desulfovibrio* sp. 6_1_46AFAA) also possessed all subunits of Rnf. Of 451 genomes of *Clostridium* sp. available in the IMG database, 392 genomes possessed all subunits of Rnf. One genome (*Clostridium* sp. KLE 1755) possessed all subunits of Ech, and it also possessed all subunits of Rnf. Of the 28 genomes of short-chain fatty acid degraders previously analyzed by Worm et al. (2014), 10 genomes possessed all subunits of Rnf. None possessed all six subunits of Ech. Of the 10 genomes of lactate fermenters previously analyzed by Weghoff et al. (2015), eight possessed all subunits of Rnf. None possessed all subunits of Ech.

**FIGURE 4:**
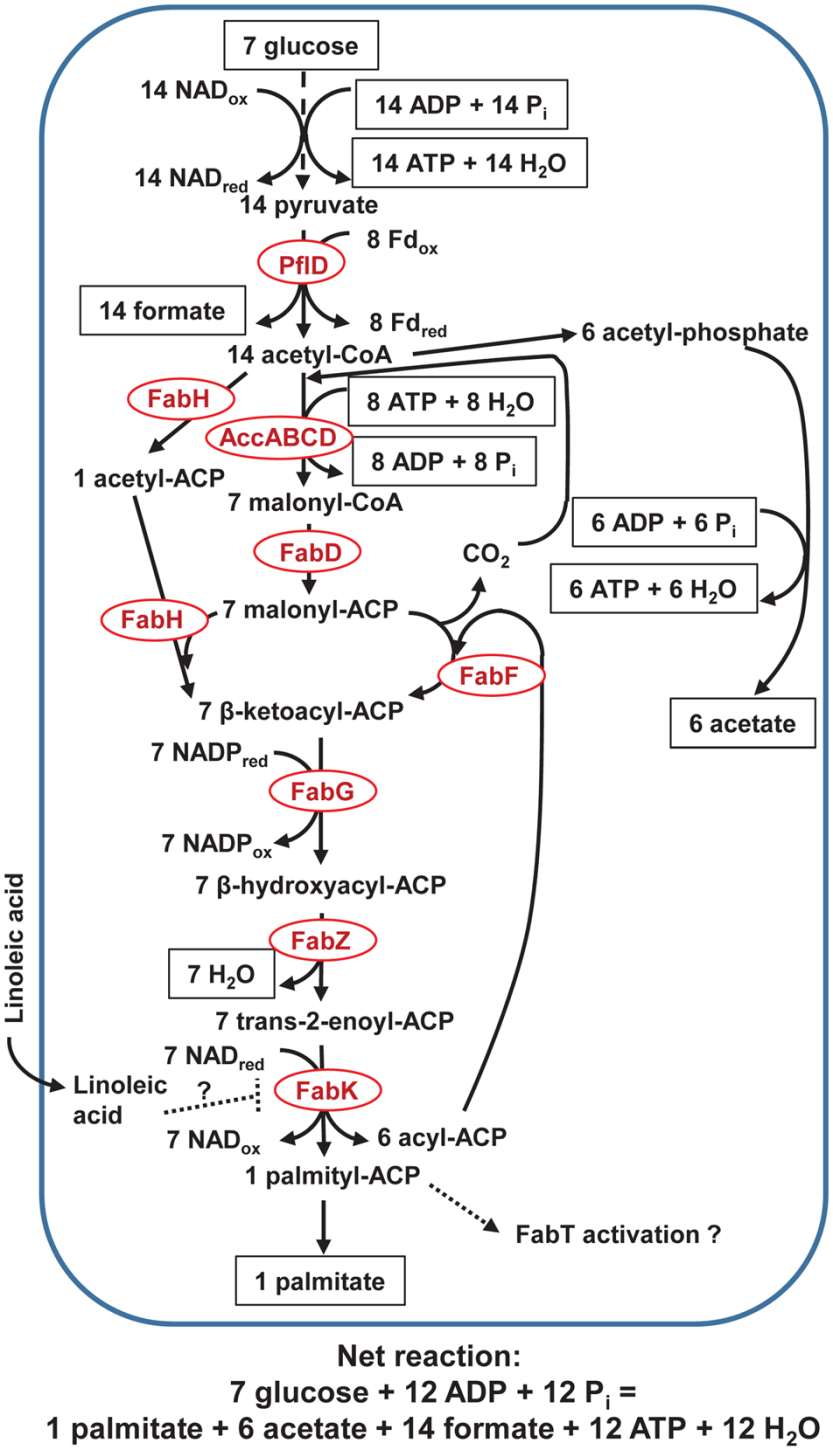
**Pathway of palmitate production in rumen butyrivibrios with PflD.** Acetate is produced to balance NAD and NADP cofactors, assuming their equivalency (see text and Supplementary Figure S2 for explanation). Only long-chain fatty acids bound to ACP might activate the Fab regulator, FabT, as indicated by a dashed arrow and documented for other non-rumen bacteria. Non-esterified linoleate is assumed to associate with or penetrate the cell membrane and hypothesized (as denoted with a dashed line) to allosterically inhibit the terminal FabK enzyme. See text for details and additional abbreviations.

Acetyl-CoA carboxylase subunits were predicted with variable results (between 36 and 61 draft genomes), and at least 60 of the 62 butyrivibrio draft genomes predicted FabD, FabF, FabG, FabZ, and FabK, whereas 41 of 62 predicted FabH (**Figure 4**). All 62 butyrivibrio draft genomes predicted the acyltransferases PlsX, PlsY, and PlsC that are needed to synthesize phosphatidic acid, the precursor for fatty acid components in bacterial membranes. No draft butyrivibrio genomes predicted transhydrogenases SthA, pntAB, or ADHFE1. As shown in Supplementary Figure S2, citrate synthase, aconitase, NADP-isocitrate dehydrogenase, malic enzyme, pyruvate kinase, pyruvate-phosphate dikinase, oxaloacetate decarboxylase, glutamate dehydrogenase, and glutamate synthase were predicted in all 62 draft genomes. The following enzymes are not shown in Supplementary Figure S2 because only 10 predicted α-ketoglutarate dehydrogenase complex (for all subunits), only four and five predicted succinate dehydrogenase (A and B subunits, respectively), and none predicted malate dehydrogenase.

## Discussion

### Electron Transport Phosphorylation Involving Both Ech and Rnf

Our genomic analysis suggests that ETP is important to rumen butyrivibrios, and it may enable them to achieve unprecedented ATP yields in fermentation of glucose to butyrate. Glucose fermentation had been long-thought to generate ATP primarily by SLP (Gottschalk, 1986; Russell, 2002; White, 2007). In prokaryotes, the highest yield possible was thought to be four ATP/glucose; this yield corresponds to fermentation of glucose to acetate and H_2_ (Russell, 2002). Higher yields could be possible in eukaryotes expressing pyrophosphate-dependent glycolytic enzymes (Mertens, 1993), but those enzymes appear to be rarely expressed in exclusion to the classic glycolytic enzymes (Müller et al., 2012).

Glucose fermentation to butyrate had been long-accepted to form three ATP by SLP (Gottschalk, 1986), even as thermodynamic evidence suggested additional ATP should be formed (Stadtman, 1966; Ungerfeld and Kohn, 2006). The recent discovery of Bcd-Etf and Rnf in *Clostridium* sp. confirmed that additional ATP could be formed from ETP (Boiangiu et al., 2005; Herrmann et al., 2008; Li et al., 2008). Specifically, Bcd-Etf generates Fd_red_ and NAD_ox_ during crotonyl-CoA reduction to butyryl-CoA, and Rnf generates ΔμNa^+^ from these cofactors. Louis and Flint (2009) suggested even more energy could be conserved during pyruvate conversion to acetyl-CoA. They suggested the existence of a PFO and transmembrane ion pump that could together produce a ΔμH^+^, but the PFO and pump were not identified.

In an analysis of rumen butyrivibrio genomes, we have identified a PFO (NifJ) and an ion pump (Ech) that serves the function suggested by Louis and Flint (2009). The presence of NifJ and Ech, along with Bcd-Etf and Rnf, could achieve yields of ∼4.5 ATP/glucose for butyrate and H_2_ formation from glucose. This yield rivals or surpasses that for fermentation of glucose to acetate and H_2_.

One pathway we propose (**Figure 1**) shows H^+^ as an electron acceptor. We propose a pathway with an unsaturated fatty acid as the electron acceptor (**Figure 2**), also, because butyrivibrios are the predominant biohydrogenating organisms in the rumen (Lourenço et al., 2010). If the electron acceptor were an unsaturated fatty acid bond, only Rnf would pump ions, but the ATP yield would be unchanged. Incubating *B. fibrisolvens* in LA decreased its intracellular ATP (Maia et al., 2010), and, all else equal, our analysis suggests that a lower ATP yield due to biohydrogenation is not responsible.

We depict a fatty acid oxidoreductase in the pathway for which unsaturated fatty acid serves as an electron acceptor. Our depiction is based on Hughes et al. (1982), who biochemically characterized and purified part of the oxidoreductase of *B. fibrisolvens* (Hughes et al., 1982). We presume the oxidoreductase is possessed by most genomes, as most butyrivibrios can biohydrogenate unsaturated fatty acids (e.g., LA; Paillard et al., 2007). However, we could not verify this presumption through a genomic search because the oxidoreductase is not found in KEGG, COG, pfam, or other databases [though a sequence of the oxidoreductase has been reported (Fukuda et al., 2007)].

As depicted, the oxidoreductase does not directly contribute to ATP synthesis by ETP. The oxidoreductase likely cannot pump ions because it appears to be a membrane-associated, not an integral, protein (Hughes et al., 1982). The oxidoreductase cannot drive Rnf or Ech to pump ions, either, because it generates only NAD_ox_ and not Fd_red_; the observed stoichiometry of NAD_red_ oxidation to rumenate reduction is 1:1 (Hunter et al., 1976) and prohibits it from generating Fd_red_. In sum, the oxidoreductase does not appear to drive ion pumping (either directly or indirectly), supporting that (1) it does not contribute to ATP synthesis and (2) ATP yield is unchanged when an unsaturated fatty acid serves as an electron acceptor in place of H^+^.

Several parts of our genomic analysis require qualification. First, although many (76%) of genomes possessed all genes for Ech and Rnf, genes for EchD and EchF were individually absent in 11.3% of butyrivibrio genomes, and they were both absent in one genome (1.6% of total). Even for organisms possessing all genes, the expression and activity of both Ech and Rnf needs to be experimentally confirmed. Some evidence of Ech activity comes from (1) the observation that butyrivibrios produce H_2_ (Hungate, 1966) and (2) our finding that most butyrivibrio genomes possessed only Ech and no other Fd-dependent hydrogenase. Some genomes possessed the HydA large subunit, part of another Fd-dependent hydrogenase, but this hydrogenase may not have activity as no other subunits were possessed.

Second, the function we ascribe to NifJ—supplying Fd_red_ to Ech or Rnf during fermentation—is putative. Historically, NifJ has been recognized for supplying Fd_red_ or flavodoxin to nitrogenase during N_2_ fixation (Shah et al., 1983; Brostedt and Nordlund, 1991), not to Ech or Rnf during fermentation. In recent experiments comparing wild-type and *nifJ* deletion mutants, however, mutants had low rates of acetate and H_2_ production during fermentation (McNeely et al., 2011; Gutekunst et al., 2014). These experiments suggest NifJ has a function in fermentation and can supply Fd_red_ to a hydrogenase (albeit the hydrogenase in those experiments was not Ech). Por, a PFO homologous to NifJ (Kletzin and Adams, 1996), was not possessed by any butyrivibrio genome.

Third, as a pyruvate formate lyase, PflD could serve as an alternative to NifJ, produce formate instead of H_2_, and lower ATP yield. ATP yield is reduced because PflD does not generate Fd_red_, which drives Ech and generates an electrochemical potential. Whereas formate oxidation by a FdhF-Hyl complex could generate Fd_red_ (Wang et al., 2013b), no butyrivibrio genome possessed the full complex.

Pyruvate formate lyase D indeed appears to serve as an alternative to NifJ in many butyrivibrios. Both H_2_ and formate are observed fermentation products of butyrivibrios (Hungate, 1966), suggesting that both PflD and NifJ are active. Across strains, large variation exists in formate vs. H_2_ production (Hungate, 1966). Within strains, also, large variation exists, with one strain (*B. fibrisolvens* 49) decreasing H_2_ and increasing formate production in response to increasing acetate in the medium (Diez-Gonzalez et al., 1999). This variation in formate vs. H_2_ production suggests that PflD and NifJ can be differentially expressed or regulated. Because the pathway with PflD yields fewer ATP than that with NifJ, this differential expression or regulation may allow butyrivibrios to modulate ATP yield. More experiments should compare the expression and activity of these enzymes and under different growth conditions.

Fourth, the methylglyoxal pathway, presuming it is complete, could serve as an alternate to a full EMP pathway and lower ATP yield. Kelly et al. (2010) suggested the methylglyoxal pathway could be important in butyrivibrios that appear to be missing a key EMP pathway enzyme (Eno). We found that 40% of butryivibrios apparently lacked Eno. Though the methylglyoxal pathway could serve as an alternative in these butyrivibrios, the ATP yield would be low (≤0.5/glucose). More work needs to determine if alternatives to the EMP pathway exist besides the methylglyoxal pathway.

Fifth, the stoichiometry of Ech, Rnf, and F_0_F_1_-ATP synthase is uncertain. Ech has been shown to pump H^+^ (Welte et al., 2010), and Rnf pumps either H^+^ or Na^+^ (Biegel and Müller, 2010; Schlegel et al., 2012; Tremblay et al., 2013). The number of ions pumped has been postulated to be two per Fd_red_ oxidized (Buckel and Thauer, 2013; Schuchmann and Müller, 2014), but this stoichiometry has not been experimentally established. This uncertainty, along with the variable stoichiometry of F_0_F_1_-ATP synthases (Nicholls and Ferguson, 2013), makes our estimate of ∼1.5 ATP/glucose formed from ETP subject to revision.

Despite its limitations, our analysis still suggests both Ech and Rnf genes are possessed by the majority of rumen butyrivibrios. Possession of both Ech and Rnf genes has been seldom documented for the same organism, much less for a groups of organisms. In their analysis of sulfate-reducing prokaryotes, Pereira et al. (2011) reported that some *Desulfovibrio* sp. possessed both Ech and Rnf. For *C. thermocellum*, Raman et al. (2011) and Rydzak et al. (2012) found some, but not all, subunits of Ech and Rnf were expressed. In their analysis of 28 genomes of short-chain fatty acid degraders, Worm et al. (2014) reported some genomes possessed several subunits of both Ech and Rnf, but their analysis did not examine some subunits (RnfA,E,F). In their analysis of 20 genomes of lactate fermenters, Weghoff et al. (2015) implied both Ech and Rnf were possessed by least one of their genomes.

We analyzed these genomes previously documented to possess both Ech and Rnf, as well as all genomes of non-butyrivibrio rumen prokaryotes. When we analyzed all *Desulfovibrio* genomes available in the IMG database, we found that five genomes (10.6% of total) possessed both Ech and Rnf. When we analyzed all *Clostridium* genomes available, we found only that only one genome (0.002% of total) possessed both Ech and Rnf. Our re-analysis of all genomes of short-chain fatty acid from Worm et al. (2014) revealed none possessed all subunits of Ech and Rnf. In our re-analysis of 10 genomes from Weghoff et al. (2015; the other 10 genomes were not explicitly identified by the authors), we did not find any genomes possessing both Ech and Rnf. In our own analysis of rumen prokaryotes, only 4.6% of non-butyrivibrio genomes were predicted to possess both Ech and Rnf. One genome belonged to the *Desulfovibrio*, and all others belonged to the Lachnospiraceae, of which butyrivibrio are members (Cotta and Forster, 2006). From present analyses, possession of both Ech and Rnf would appear rare outside of the butyrivibrios, related organisms, and a few sulfate-reducers.

Rather than being possessed together, Ech and Rnf are usually possessed separately and appear to substitute in function. This point is made by the reductive acetogens. Three model acetogens (*A. woodi, C. ljungdahlii, M. thermoacetica*) carry out similar pathways of reductive acetogenesis, except the former 2 possess Rnf and the lattermost possesses Ech (Buckel and Thauer, 2013; Schuchmann and Müller, 2014).

Although most butyrivibrio genomes possessed all subunits of Ech and Rnf, some genomes did not, and these exceptions may help in discriminating between butyrivibrio species. Butyrivibrios make up a genetically diverse group (Cotta and Forster, 2006), but few properties are related to phylogenetic position and can be used to discriminate between different species or phylotypes. Some discriminatory properties previously suggested include butyrate kinase activity, lipase activity, products of linoleate biohydrogenation, and sensitivity to linoleate (Paillard et al., 2007). Other suggested discriminitory properties, such as substrate utilization and cell fatty acid composition, have been criticized as inadequate (Cotta and Forster, 2006; Willems and Collins, 2009). We suggest that absence of Ech subunits may serve as another discriminatory property. Absence of EchD may be a useful property, for example, as all six genomes classified as *B. fibrisolvens* were missing EchD alone; this absence was observed in only one other genome (an unclassified *Butyrivibrio* sp.). Absence of EchD would be even more meaningful if later associated with a phenotype (e.g., absent or altered Ech activity). More discriminatory properties might emerge once our results can be compared to a full a phylogenetic tree of Hungate 1000 strains (full-length 16S rDNA sequences are not yet available).

The possession of both Ech and Rnf by most rumen butyrivibrios suggests a functional importance to this group. Likely, it permits high ATP yields from fermentation and confers an energetic advantage. Such an energetic advantage could help support the metabolic flexibility observed for members of this group. Butyrivibrio strains are capable of degrading an unusually wide range of carbohydrates, and many can degrade protein (Hungate, 1966; Stewart et al., 1997; Cotta and Forster, 2006). Such metabolic flexibility comes at the cost of producing a host of degradative enzymes, but this cost may be offset by a high ATP yield from ETP. If high ATP yields are not required (e.g., during growth limitation), upregulation of PflD, downregulation of NifJ, and upregulation of methylglyoxal pathway enzymes could lower ATP yield (e.g., to prevent energy spilling; Hackmann and Firkins, 2015). The unique combination of genes supporting ETP makes butyrivibrio strains attractive for further study as model anaerobic bacteria. Further, the majority of rumen prokaryotes possess at least Rnf or Ech, suggesting ETP has importance in the rumen beyond just the butyrivibrios and merits further study in itself.

### Hypothesis for Varying Linoleic Acid Toxicity by the Butyrivibrios

**Figure 4** balances reducing equivalents using PflD (**Figure 3**). Although Fd_red_ produced from NifJ (**Figure 1**) could be reoxidized using a cytosolic FeFe hydrogenase as depicted in Louis and Flint (2009), we could find little evidence for such a role in the butyrivibrio draft genomes. The NAD_red_ needed for synthesis of palmitate required extra acetate production because butyrate production would have reoxidized that NAD_red_ (**Figures 1** and **3**); we note that acetate could be reused for those butyrivibrios producing butyrate through butyryl-CoA acetyl-CoA transferase (Diez-Gonzalez et al., 1999). The ATP loss in **Figure 4** compared with **Figure 1** through **Figure 3** (if converted to seven moles of glucose) is consistent with the expectation of considerable ATP sparing if exogenous fatty acids are incorporated in rumen bacterial membranes (Wu and Palmquist, 1991; Vlaeminck et al., 2006).

The ATP yield per glucose can be maintained with moderate rumenate reductase activity during biohydrogenation (**Figure 2**), but a high dose of LA increases the lag for growth in *B. fibrisolvens* JW11 (Maia et al., 2010). In contrast with an apparent constitutive expression of the cluster of genes used in butyrate production (Asanuma et al., 2005), unsaturated fatty acids probably increase expression of rumenate reductase (Fukuda et al., 2007) for *B. fibrisolvens*. Biohydrogenation activity was assumed to depend on provision of reducing equivalents from the EMP pathway (Kim, 2003). Our hypothesis builds on the foundations that bolus-dosed LA disrupts synthesis of fatty acids needed to generate membranes during growth of the butyrivibrios because of competition for acetyl-CoA and reducing equivalents.

The butyrivibrios use the standard enzymes for fatty acid (**Figure 4**) and phosphatidic acid synthesis for membrane components (Parsons and Rock, 2013). They discussed that FabH often has species-specific affinity for priming units (often for unsaturated or branched-chain fatty acids), but FabH was only predicted in 66% of the butyrivibrio draft genomes. For mixed rumen bacteria, synthesis of *iso* and *anteiso* fatty acids (which increase fluidity) is thought to be fixed within species, whereas synthesis of even- and odd-chain fatty acids is a function of availability of primers (Vlaeminck et al., 2006).

Jenkins et al. (2008) extended the butyrivibrio taxonomic grouping of Paillard et al. (2007) into three groups. The *B. fibrisolvens* and *Pseudobutyrivibrio* clades stop biohydrogenation of LA at VA (i.e., the VA1 group) and consume acetate to produce butyrate through a butyryl-CoA acetyl-CoA transferase. The *B. hungatei* clade also stops biohydrogenation at VA (i.e., the VA2 group). The *B. proteoclasticus* clade biohydrogenates LA fully to SA (i.e., the SA group). Compared with the VA1 group, the VA2 and SA groups are more sensitive to LA, and both produce butyrate through butyrate kinase. Consistent with this pattern, Kopecný et al. (2003) grouped >40 isolates of butyrivibrios based on fatty acid profile. *B. fibrisolvens, P. ruminis*, and *P. xylanivorans* in the VA1 group have higher 16:0 and have no *iso*-16:0 or *anteiso*-17:0; in contrast, *B. hungatei* (VA2 group) and *B. proteoclasticus* (SA group) have much lower 16:0 and higher *anteiso*-17:0, and *B. proteoclasticus* has particularly high *iso*-14:0 and *anteiso*-15:0 concentration. Because of the increased branched (*iso* and *anteiso*) fatty acids, VA2 and especially SA members should be more sensitive to the fluidization from LA permeabilizing their membranes, which are conspicuously thin (Maia et al., 2010). In response, *de novo* synthesis of saturated fatty acids from acetyl-CoA might increase to counter the increased membrane fluidity.

In the rumen, the butyrivibrios must be in close proximity to their substrate, plant hemicelluloses (Kelly et al., 2010). Although not studied specifically with butyrivibrios, mixed particulate-phase bacteria incorporated LA at about double that of fluid-phase bacteria harvested from the rumen after soybean oil was fed, and non-esterified fatty acids were internalized (Bauchart et al., 1990). Both α- or β-oxidation of acyl-CoA is minimal in mixed ruminal bacteria (Wu and Palmquist, 1991). Those authors discussed the likelihood that fatty acids <14 carbons were elongated, but exogenous palmitic acid and SA replaced *de novo* synthesis with little net change in fatty acid composition after dosing fat containing LA. Because butyrivibrios have little 18-carbon fatty acids in their membranes (Kopecný et al., 2003) compared with the much larger percentage (>40% SA) in composites of mixed rumen bacteria (Or-Rashid et al., 2007), the limited β-oxidation of SA to palmitic acid might render dietary 18-carbon fatty acids of little benefit to butyrivibrios.

Our hypothesis assumes that *de novo* synthesis of palmitate would be feedback-interrupted by intracellular LA (**Figure 4**). Because cultures must be growing to biohydrogenate fatty acids at concentrations that are toxic to stationary phase cultures (McKain et al., 2010), fatty acid synthesis presumably is induced in coordination with growth. The series of enzymes in **Figure 4** (from AccABCD to FabK) is the same as that repressed by FabT in other bacteria (Parsons and Rock, 2013). However, they noted that FabT only has a moderating effect. We could not verify if FabT is possessed by butyrivibrios because FabT is not found in KEGG, COG, pfam, or other databases. Non-esterified LA and other long chain unsaturated fatty acids—but not saturated fatty acids—allosterically inhibited FabI (catalyzing the same reaction as FabK) and inhibited ^14^C-acetate incorporation into membrane lipids of *Staphylococcus aureus* (Zheng et al., 2005). Although FabK is a flavoprotein (unlike FabI), LA inhibition was a result of both its binding to the enzyme and the enzyme-reduced cofactor complex. Because FabI rate-limits fatty acid synthesis, ACP-bound intermediates accumulated from sustained acetyl-CoA carboxylase activity (Cronan, 2014). Consequently, allosteric inhibition by non-esterified LA (i.e., not bound to ACP) of FabK might increase pooling of acyl-ACP intermediates in butyrivibrios after LA concentration at the cell membrane exceeds their membrane-associated biohydrogenation capacity and is internalized.

Although we assumed reduced nucleotide cofactors are interconvertible in **Figure 4**, cellular mechanisms to balance reducing equivalents might explain why lactate accentuated LA toxicity in butyrivibrios (Paillard et al., 2007). Cytosolic SthA or membrane-associated PntAB transhydrogenases (Fuhrer and Sauer, 2009) were not recovered from the butyrivibrio draft genomes, and NfnAB (Buckel and Thauer, 2013) has not yet been annotated by KEGG or COG for our search. Fluxing of acetyl-CoA though NADP-isocitrate dehydrogenase to α-ketoglutarate would produce NADP_red_, but some NADP_red_ might be reoxidized to synthesize glutamate for amino acid biosynthesis (Supplementary Figure S2). *C. thermocellum* produces NADP_red_ through malic enzyme and malate dehydrogenase cycling (Burton and Martin, 2012). All of those enzymes (Supplementary Figure S2) were uniformly in the butyrivibrio draft genomes except for the enzyme critical to complete a cycle, malate dehydrogenase (uniformly absent). However, other enzymes involved in pyruvate metabolism (especially oxaloacetate decarboxylase and pyruvate-phosphate dikinase) should be investigated for potential to substitute for lack of Eno (see earlier discussion) in production of oxaloacetate for anabolic reactions such as amino acid synthesis. β-hydroxybutyrl-CoA dehydrogenase was proposed as a major supplier of NADP_red_ for anabolic reactions in *B. fibrisolvens* D1 (Miller and Jenesel, 1979). Cycling of forward (oxidizing NAD_red_ when producing β-hydroxybutyrl-CoA) and backward (reducing NADP_ox_ when producing acetoacetyl-CoA) reactions would supply NADP_red_ for fatty acid synthesis and also explain how the acetoacetyl-CoA pools were maintained while other acyl-CoA pools used in butyrate production were dramatically depleted after LA dosing (Maia et al., 2010). Because of reverse Ldh activity after a large dose of lactate, a large increase in NAD_red_ could thermodynamically decrease this cycling to produce NADP_red_ for FabG (**Figure 4**).

Further biochemical characterization is needed to understand how butyrivibrios respond metabolically to high LA challenge coinciding with high carbohydrate availability to circumvent lactate-tolerant bacteria from biohydrogenating LA through alternate *trans*-10 18:1 pathways (McKain et al., 2010). However, the extra ATP yield from mechanisms described in **Figures 1** and **2** likely help the butyrivibrios recover from conditions in which LA exceeds biohydrogenation capacity.

## Conflict of Interest Statement

The authors declare that the research was conducted in the absence of any commercial or financial relationships that could be construed as a potential conflict of interest.

## Abbreviations

ACP: acyl carrier protein
Bcd-Etf: butyryl-CoA dehydrogenase/electron transferring flavoprotein
COG: clusters of orthologous groups
Ech: *Escherichia coli* hydrogenase-3-type hydrogenase
Eha: energy-converting hydrogenase A
Ehb: energy-converting hydrogenase B
EMP: Embden–Meyerhof–Parnas
Eno: enolase
ETP: electron transport phosphorylation
Fab: fatty acid biosynthesis enzymes
Fd: ferredoxin
Fdh: formate dehydrogenase
Fd_ox_: oxidized Fd
Fd_red_: reduced Fd
Glo: glyoxalase
Hyc: hydrogenase 3
Hyd: ferredoxin hydrogenase
Hyl: hydrogenase-like protein
KO: Kyoto Encyclopedia of Genes and Genomes orthology
LA: linoleic acid
Ldh: lactate dehydrogenase
Mbh: membrane-bound hydrogenase
MsgA: methylglyoxal synthase
Mvh/Hdr: methyl viologen hydrogenase/heterodisulfide reductase
NAD: nicotinamide adenine dinucleotide
NAD_ox_: oxidized NAD
NAD_red_: reduced NAD
NifJ: nitrogen fixation J
PflD: pyruvate formate lyase D
Por: pyruvate ferredoxin oxidoreductase
Rnf: *Rhodobacter* nitrogen fixation
SA: stearic acid
SLP: substrate-level phosphorylation
VA: *trans*-11 vaccenic acid
ΔμH^+^: proton electrochemical potential
ΔμNa^+^: sodium electrochemical potential
