# Supplementary material for: Electron transport phosphorylation in rumen butyrivibrios: unprecedented ATP yield for glucose fermentation to butyrate
Source: Front Microbiol. 2015 Jun 24;6:622. doi: 10.3389/fmicb.2015.00622 (PMC4478896; doi:10.3389/fmicb.2015.00622)
Supplement: Supplementary file 1 [file Table_1.DOCX]

**Table S1. Predicted number of genes for subunits of RnfABCDEG and EchABCDEF for rumen bacterial and archaeal genomes in the Hungate 1000 project.^1^**

|  | Rnf | | | | | |  | Ech | | | | | |
| --- | --- | --- | --- | --- | --- | --- | --- | --- | --- | --- | --- | --- | --- |
| Genome name | A | B | C | D | E | G |  | A | B | C | D | E | F |
| Acidaminococcus fermentans pGA-4 | 0 | 1 | 1 | 1 | 1 | 0 |  | 0 | 0 | 0 | 0 | 0 | 0 |
| Bacteroides sp. AR20 | 1 | 1 | 1 | 1 | 1 | 1 |  | 0 | 0 | 0 | 0 | 0 | 0 |
| Bacteroides sp. AR29 | 1 | 1 | 1 | 1 | 1 | 1 |  | 0 | 0 | 0 | 0 | 0 | 0 |
| Bacteroides sp. Ga6A1 | 1 | 1 | 1 | 1 | 1 | 1 |  | 0 | 0 | 0 | 0 | 0 | 0 |
| Bacteroides sp. Ga6A2 | 1 | 1 | 1 | 1 | 1 | 1 |  | 0 | 0 | 0 | 0 | 0 | 0 |
| Bacteroides thetaiotaomicron KPPR-3 | 1 | 1 | 1 | 1 | 1 | 1 |  | 0 | 0 | 0 | 0 | 0 | 0 |
| Basfia succiniciproducens DSM 22022 | 1 | 1 | 1 | 1 | 1 | 1 |  | 0 | 0 | 0 | 0 | 0 | 0 |
| Basfia succiniciproducens KPR-2 | 1 | 1 | 1 | 1 | 1 | 1 |  | 0 | 0 | 0 | 0 | 0 | 0 |
| Blautia schinkii DSM 10518 | 1 | 1 | 3 | 2 | 1 | 1 |  | 0 | 0 | 0 | 0 | 0 | 0 |
| Blautia sp. SF-50 | 1 | 1 | 2 | 1 | 1 | 1 |  | 0 | 0 | 0 | 0 | 0 | 0 |
| Blautia wexlerae AGR2146 | 1 | 1 | 2 | 1 | 1 | 1 |  | 0 | 0 | 0 | 0 | 0 | 0 |
| Butyrivibrio fibrisolvens AB2020 | 1 | 1 | 1 | 1 | 1 | 1 |  | 1 | 1 | 1 | 0 | 1 | 1 |
| Butyrivibrio fibrisolvens FE2007 | 1 | 1 | 1 | 1 | 1 | 1 |  | 1 | 1 | 1 | 0 | 1 | 1 |
| Butyrivibrio fibrisolvens MD2001 | 1 | 1 | 1 | 1 | 1 | 1 |  | 1 | 1 | 1 | 0 | 1 | 1 |
| Butyrivibrio fibrisolvens ND3005 | 1 | 1 | 1 | 1 | 1 | 1 |  | 1 | 1 | 1 | 0 | 1 | 1 |
| Butyrivibrio fibrisolvens WTE3004 | 1 | 1 | 1 | 1 | 1 | 1 |  | 1 | 1 | 1 | 0 | 1 | 1 |
| Butyrivibrio fibrisolvens YRB2005 | 1 | 1 | 1 | 1 | 1 | 1 |  | 1 | 1 | 1 | 0 | 1 | 1 |
| Butyrivibrio hungatei NK4A153 | 1 | 1 | 1 | 1 | 1 | 1 |  | 1 | 1 | 1 | 1 | 1 | 1 |
| Butyrivibrio hungatei XBD2006 | 1 | 1 | 1 | 1 | 1 | 1 |  | 1 | 1 | 1 | 1 | 1 | 1 |
| Butyrivibrio proteoclasticus FD2007 | 1 | 1 | 1 | 1 | 1 | 1 |  | 1 | 1 | 1 | 1 | 1 | 1 |
| Butyrivibrio proteoclasticus P6B7 | 1 | 1 | 1 | 1 | 1 | 1 |  | 1 | 1 | 1 | 1 | 1 | 1 |
| Butyrivibrio sp. AC2005 | 1 | 1 | 1 | 1 | 1 | 1 |  | 1 | 1 | 1 | 1 | 1 | 1 |
| Butyrivibrio sp. AD3002 | 1 | 1 | 1 | 1 | 1 | 1 |  | 1 | 1 | 1 | 1 | 1 | 1 |
| Butyrivibrio sp. AE2005 | 1 | 1 | 1 | 1 | 1 | 1 |  | 1 | 1 | 1 | 1 | 1 | 1 |
| Butyrivibrio sp. AE2015 | 1 | 1 | 1 | 1 | 1 | 1 |  | 1 | 1 | 1 | 1 | 1 | 1 |
| Butyrivibrio sp. AE2032 | 1 | 1 | 1 | 1 | 1 | 1 |  | 1 | 1 | 1 | 1 | 1 | 1 |
| Butyrivibrio sp. AE3003 | 1 | 1 | 1 | 1 | 1 | 1 |  | 1 | 1 | 1 | 1 | 1 | 1 |
| Butyrivibrio sp. AE3004 | 1 | 1 | 1 | 1 | 1 | 1 |  | 1 | 1 | 1 | 1 | 1 | 1 |
| Butyrivibrio sp. AE3006 | 1 | 1 | 1 | 1 | 1 | 1 |  | 1 | 1 | 1 | 1 | 1 | 1 |
| Butyrivibrio sp. AE3009 | 1 | 1 | 1 | 1 | 1 | 1 |  | 1 | 1 | 1 | 1 | 1 | 1 |
| Butyrivibrio sp. FC2001 | 1 | 1 | 1 | 1 | 1 | 1 |  | 1 | 1 | 1 | 1 | 1 | 1 |
| Butyrivibrio sp. FCS006 | 1 | 1 | 1 | 1 | 1 | 1 |  | 1 | 1 | 1 | 1 | 1 | 1 |
| Butyrivibrio sp. FCS014 | 1 | 1 | 1 | 1 | 1 | 1 |  | 1 | 1 | 1 | 1 | 1 | 1 |
| Butyrivibrio sp. INlla14 | 1 | 1 | 1 | 1 | 1 | 1 |  | 1 | 1 | 1 | 1 | 1 | 1 |
| Butyrivibrio sp. INlla16 | 1 | 1 | 1 | 1 | 1 | 1 |  | 1 | 1 | 1 | 1 | 1 | 1 |
| Butyrivibrio sp. INlla18 | 1 | 1 | 1 | 1 | 1 | 1 |  | 1 | 1 | 1 | 1 | 1 | 1 |
| Butyrivibrio sp. INlla21 | 1 | 1 | 1 | 1 | 1 | 1 |  | 1 | 1 | 1 | 1 | 1 | 1 |
| Butyrivibrio sp. LB2008 | 1 | 1 | 1 | 1 | 1 | 1 |  | 1 | 1 | 1 | 1 | 1 | 1 |
| Butyrivibrio sp. LC3010 | 1 | 1 | 1 | 1 | 1 | 1 |  | 1 | 1 | 1 | 1 | 1 | 1 |
| Butyrivibrio sp. MB2005 | 1 | 1 | 1 | 1 | 1 | 1 |  | 1 | 1 | 1 | 1 | 1 | 1 |
| Butyrivibrio sp. MC2013 | 1 | 1 | 1 | 1 | 1 | 1 |  | 1 | 1 | 1 | 1 | 1 | 0 |
| Butyrivibrio sp. MC2021 | 1 | 1 | 1 | 1 | 1 | 1 |  | 1 | 1 | 1 | 1 | 1 | 1 |
| Butyrivibrio sp. NC2002 | 1 | 1 | 1 | 1 | 1 | 1 |  | 1 | 1 | 1 | 1 | 1 | 1 |
| Butyrivibrio sp. NC2007 | 1 | 1 | 1 | 1 | 1 | 1 |  | 1 | 1 | 1 | 1 | 1 | 1 |
| Butyrivibrio sp. NC3005 | 1 | 1 | 1 | 1 | 1 | 1 |  | 0 | 0 | 0 | 0 | 0 | 0 |
| Butyrivibrio sp. OB235 | 1 | 1 | 1 | 1 | 1 | 1 |  | 1 | 1 | 1 | 1 | 1 | 1 |
| Butyrivibrio sp. Su6 | 1 | 1 | 1 | 1 | 1 | 1 |  | 1 | 1 | 1 | 1 | 1 | 1 |
| Butyrivibrio sp. TB | 1 | 1 | 1 | 1 | 1 | 1 |  | 1 | 1 | 1 | 0 | 1 | 1 |
| Butyrivibrio sp. VCB2001 | 1 | 1 | 1 | 1 | 1 | 1 |  | 1 | 1 | 1 | 1 | 1 | 1 |
| Butyrivibrio sp. VCB2006 | 1 | 1 | 1 | 1 | 1 | 1 |  | 1 | 1 | 1 | 1 | 1 | 1 |
| Butyrivibrio sp. VCD2006 | 1 | 1 | 1 | 1 | 1 | 1 |  | 1 | 1 | 1 | 1 | 1 | 1 |
| Butyrivibrio sp. WCD2001 | 1 | 1 | 1 | 1 | 1 | 1 |  | 1 | 1 | 1 | 1 | 1 | 1 |
| Butyrivibrio sp. WCD3002 | 1 | 1 | 1 | 1 | 1 | 1 |  | 1 | 1 | 1 | 1 | 1 | 1 |
| Butyrivibrio sp. WCE2006 | 1 | 1 | 1 | 1 | 1 | 1 |  | 1 | 1 | 1 | 1 | 1 | 1 |
| Butyrivibrio sp. XBB1001 | 1 | 1 | 1 | 1 | 1 | 1 |  | 1 | 1 | 1 | 1 | 1 | 1 |
| Butyrivibrio sp. XPD2002 | 1 | 1 | 1 | 1 | 1 | 1 |  | 1 | 1 | 1 | 1 | 1 | 1 |
| Butyrivibrio sp. XPD2006 | 1 | 1 | 1 | 1 | 1 | 1 |  | 1 | 1 | 1 | 1 | 1 | 1 |
| Butyrivibrio sp. YAB3001 | 1 | 1 | 1 | 1 | 1 | 1 |  | 1 | 1 | 1 | 1 | 1 | 1 |
| Clostridiales bacterium NK3B98 | 1 | 1 | 1 | 0 | 1 | 1 |  | 0 | 0 | 0 | 0 | 0 | 0 |
| Clostridiales bacterium WTE2008 | 1 | 1 | 1 | 0 | 1 | 1 |  | 0 | 0 | 0 | 0 | 0 | 0 |
| Clostridium aerotolerans DSM 5434 | 1 | 0 | 1 | 1 | 1 | 1 |  | 1 | 1 | 1 | 1 | 1 | 0 |
| Clostridium aminophilum F | 1 | 1 | 1 | 1 | 1 | 1 |  | 0 | 0 | 0 | 0 | 0 | 0 |
| Clostridium aminophilum KH1P1 | 1 | 1 | 1 | 1 | 1 | 1 |  | 0 | 0 | 0 | 0 | 0 | 0 |
| Clostridium beijerinckii HUN142 | 1 | 1 | 1 | 1 | 1 | 1 |  | 0 | 0 | 0 | 0 | 0 | 0 |
| Clostridium cadaveris AGR2141 | 1 | 1 | 1 | 1 | 1 | 1 |  | 0 | 0 | 0 | 0 | 0 | 0 |
| Clostridium clostidiforme ATCC 25537 | 1 | 1 | 1 | 1 | 1 | 1 |  | 0 | 0 | 0 | 0 | 0 | 0 |
| Clostridium clostridioforme AGR2157 | 1 | 1 | 1 | 1 | 1 | 1 |  | 0 | 0 | 0 | 0 | 0 | 0 |
| Clostridium glycolicum KPPR-9 | 1 | 1 | 1 | 1 | 1 | 1 |  | 0 | 0 | 0 | 0 | 0 | 0 |
| Clostridium lundense DSM 17049 | 1 | 1 | 1 | 1 | 1 | 1 |  | 0 | 0 | 0 | 0 | 0 | 0 |
| Clostridium mangenotii LM2 | 1 | 1 | 1 | 1 | 1 | 1 |  | 0 | 0 | 0 | 0 | 0 | 0 |
| Clostridium paraputrificum AGR2156 | 1 | 1 | 1 | 1 | 1 | 1 |  | 0 | 0 | 0 | 0 | 0 | 0 |
| Clostridium polysaccharolyticum DSM1801 | 1 | 1 | 1 | 1 | 1 | 1 |  | 0 | 0 | 0 | 0 | 0 | 0 |
| Denitrobacterium detoxificans DSM 21843 | 0 | 0 | 0 | 0 | 0 | 0 |  | 0 | 1 | 1 | 0 | 1 | 0 |
| Desulfovibrio desulfuricans DSM 7057 | 1 | 1 | 1 | 1 | 1 | 1 |  | 1 | 1 | 1 | 1 | 1 | 1 |
| Dorea longicatena AGR2136 | 1 | 1 | 1 | 1 | 1 | 1 |  | 0 | 0 | 0 | 0 | 0 | 0 |
| Dorea sp. AGR2135 | 1 | 1 | 1 | 1 | 1 | 1 |  | 0 | 0 | 0 | 0 | 0 | 0 |
| Enterobacter sp. KPR-6 | 1 | 1 | 1 | 1 | 1 | 1 |  | 0 | 0 | 0 | 0 | 0 | 0 |
| Enterococcus mundtii C2 | 0 | 0 | 1 | 1 | 0 | 0 |  | 0 | 0 | 0 | 0 | 0 | 0 |
| Enterococcus sp. KPPR-6 | 1 | 1 | 2 | 2 | 1 | 1 |  | 0 | 0 | 0 | 0 | 0 | 0 |
| Erysipelotrichaceae bacterium NK3D112 | 1 | 0 | 1 | 1 | 1 | 1 |  | 0 | 0 | 0 | 0 | 0 | 0 |
| Escherichia coli PA-3 | 1 | 1 | 1 | 1 | 1 | 1 |  | 0 | 0 | 0 | 0 | 0 | 0 |
| Eubacterium cellulosolvens LD2006 | 1 | 1 | 1 | 1 | 1 | 1 |  | 1 | 0 | 1 | 0 | 1 | 1 |
| Eubacterium oxidoreducens DSM 3217 | 1 | 1 | 1 | 1 | 1 | 1 |  | 0 | 0 | 0 | 0 | 0 | 0 |
| Eubacterium ruminatium HUN269 | 1 | 1 | 1 | 0 | 1 | 1 |  | 0 | 0 | 0 | 0 | 0 | 0 |
| Eubacterium sp. AB3007 | 1 | 1 | 1 | 1 | 1 | 1 |  | 0 | 0 | 0 | 0 | 0 | 0 |
| Fusobacterium necrophorum HUN048 | 1 | 1 | 1 | 1 | 1 | 1 |  | 0 | 0 | 0 | 0 | 0 | 0 |
| Lachnobacterium bovis AE2004 | 1 | 1 | 1 | 1 | 1 | 1 |  | 0 | 0 | 0 | 0 | 0 | 0 |
| Lachnobacterium bovis C6A12 | 1 | 1 | 1 | 1 | 1 | 1 |  | 0 | 0 | 0 | 0 | 0 | 0 |
| Lachnobacterium bovis DSM 14045 | 1 | 1 | 1 | 1 | 1 | 1 |  | 0 | 0 | 0 | 0 | 0 | 0 |
| Lachnobacterium bovis NK4B19 | 1 | 1 | 1 | 1 | 1 | 1 |  | 0 | 0 | 0 | 0 | 0 | 0 |
| Lachnobacterium bovis S1b | 1 | 1 | 1 | 1 | 1 | 1 |  | 0 | 0 | 0 | 0 | 0 | 0 |
| Lachnospira multipara ATCC 19207 | 1 | 1 | 1 | 1 | 1 | 1 |  | 1 | 1 | 1 | 1 | 0 | 1 |
| Lachnospira multipara LB2003 | 1 | 1 | 1 | 1 | 1 | 1 |  | 1 | 1 | 1 | 1 | 0 | 1 |
| Lachnospira multipara MC2003 | 1 | 1 | 1 | 1 | 1 | 1 |  | 1 | 1 | 1 | 1 | 0 | 1 |
| Lachnospiraceae bacterium A10 | 1 | 1 | 1 | 1 | 1 | 1 |  | 0 | 0 | 0 | 0 | 0 | 0 |
| Lachnospiraceae bacterium AB2028 | 0 | 0 | 0 | 0 | 0 | 0 |  | 1 | 1 | 1 | 1 | 1 | 1 |
| Lachnospiraceae bacterium AC2012 | 1 | 1 | 1 | 1 | 1 | 1 |  | 0 | 0 | 0 | 0 | 0 | 0 |
| Lachnospiraceae bacterium AC2014 | 1 | 1 | 1 | 1 | 0 | 1 |  | 1 | 1 | 1 | 1 | 1 | 1 |
| Lachnospiraceae bacterium AC2028 | 1 | 1 | 1 | 1 | 1 | 1 |  | 0 | 0 | 0 | 0 | 0 | 0 |
| Lachnospiraceae bacterium AC2029 | 1 | 1 | 1 | 0 | 1 | 1 |  | 0 | 0 | 0 | 0 | 0 | 0 |
| Lachnospiraceae bacterium AC3007 | 1 | 1 | 1 | 1 | 1 | 1 |  | 1 | 1 | 1 | 1 | 1 | 1 |
| Lachnospiraceae bacterium AD3010 | 1 | 1 | 1 | 1 | 1 | 1 |  | 1 | 1 | 1 | 1 | 1 | 1 |
| Lachnospiraceae bacterium C10 | 1 | 1 | 1 | 1 | 1 | 1 |  | 0 | 0 | 0 | 0 | 0 | 0 |
| Lachnospiraceae bacterium C6A11 | 1 | 1 | 1 | 0 | 1 | 1 |  | 0 | 0 | 0 | 0 | 0 | 0 |
| Lachnospiraceae bacterium C7 | 1 | 1 | 1 | 1 | 1 | 1 |  | 0 | 0 | 0 | 0 | 0 | 0 |
| Lachnospiraceae bacterium FD2005 | 1 | 1 | 1 | 1 | 1 | 1 |  | 0 | 0 | 0 | 0 | 0 | 0 |
| Lachnospiraceae bacterium FE2018 | 1 | 1 | 1 | 1 | 1 | 1 |  | 1 | 1 | 1 | 1 | 1 | 1 |
| Lachnospiraceae bacterium G11 | 1 | 1 | 1 | 1 | 1 | 1 |  | 0 | 0 | 0 | 0 | 0 | 0 |
| Lachnospiraceae bacterium G41 | 1 | 1 | 1 | 1 | 1 | 1 |  | 0 | 0 | 0 | 0 | 0 | 0 |
| Lachnospiraceae bacterium KH1P17 | 1 | 1 | 1 | 1 | 1 | 1 |  | 1 | 1 | 1 | 1 | 1 | 1 |
| Lachnospiraceae bacterium MA2020 | 1 | 1 | 1 | 1 | 1 | 1 |  | 0 | 0 | 0 | 0 | 0 | 0 |
| Lachnospiraceae bacterium MC2017 | 1 | 1 | 1 | 1 | 1 | 1 |  | 1 | 1 | 1 | 1 | 1 | 1 |
| Lachnospiraceae bacterium MD2004 | 1 | 1 | 1 | 1 | 1 | 1 |  | 0 | 0 | 0 | 0 | 0 | 0 |
| Lachnospiraceae bacterium NC2004 | 0 | 0 | 0 | 0 | 0 | 0 |  | 1 | 0 | 1 | 1 | 1 | 1 |
| Lachnospiraceae bacterium NC2008 | 1 | 1 | 1 | 1 | 1 | 1 |  | 0 | 0 | 0 | 0 | 0 | 0 |
| Lachnospiraceae bacterium ND2006 | 1 | 1 | 1 | 0 | 1 | 1 |  | 0 | 0 | 0 | 0 | 0 | 0 |
| Lachnospiraceae bacterium NK3A20 | 1 | 1 | 2 | 1 | 1 | 1 |  | 1 | 1 | 1 | 1 | 1 | 1 |
| Lachnospiraceae bacterium NK4A136 | 1 | 1 | 1 | 1 | 1 | 1 |  | 0 | 0 | 0 | 0 | 0 | 0 |
| Lachnospiraceae bacterium NK4A144 | 1 | 1 | 1 | 1 | 1 | 1 |  | 1 | 1 | 1 | 1 | 1 | 1 |
| Lachnospiraceae bacterium NK4A179 | 0 | 0 | 0 | 0 | 0 | 0 |  | 1 | 1 | 1 | 1 | 1 | 1 |
| Lachnospiraceae bacterium P6A3 | 1 | 1 | 1 | 1 | 1 | 1 |  | 0 | 0 | 0 | 0 | 0 | 0 |
| Lachnospiraceae bacterium P6B14 | 1 | 1 | 1 | 1 | 1 | 1 |  | 1 | 1 | 1 | 1 | 1 | 1 |
| Lachnospiraceae bacterium RM5 | 1 | 1 | 1 | 1 | 1 | 1 |  | 0 | 0 | 0 | 0 | 0 | 0 |
| Lachnospiraceae bacterium V9D3004 | 0 | 0 | 0 | 0 | 0 | 0 |  | 1 | 1 | 1 | 1 | 1 | 1 |
| Lachnospiraceae bacterium XBB1006 | 1 | 1 | 1 | 1 | 1 | 1 |  | 0 | 0 | 0 | 0 | 0 | 0 |
| Lachnospiraceae bacterium XBB2008 | 1 | 1 | 1 | 1 | 1 | 1 |  | 0 | 0 | 0 | 0 | 0 | 0 |
| Lachnospiraceae bacterium XBD2001 | 1 | 1 | 1 | 1 | 1 | 1 |  | 1 | 1 | 1 | 1 | 1 | 1 |
| Lachnospiraceae bacterium XPB1003 | 0 | 0 | 0 | 0 | 0 | 0 |  | 1 | 1 | 1 | 1 | 1 | 1 |
| Lachnospiraceae bacterium YSD2013 | 1 | 1 | 1 | 1 | 1 | 1 |  | 0 | 0 | 0 | 0 | 0 | 0 |
| Megasphaera elsdenii T81 | 1 | 1 | 1 | 1 | 1 | 1 |  | 0 | 0 | 0 | 0 | 0 | 0 |
| Megasphaera elsdenii YE34 | 1 | 1 | 1 | 1 | 1 | 1 |  | 0 | 0 | 0 | 0 | 0 | 0 |
| Methanomicrobium mobile BP, DSM 1539 | 0 | 0 | 0 | 0 | 0 | 0 |  | 0 | 0 | 1 | 1 | 0 | 0 |
| Methanosarcina sp. DSM 11855 | 0 | 0 | 0 | 0 | 0 | 0 |  | 1 | 1 | 2 | 1 | 1 | 1 |
| Oribacterium sp. FC2011 | 1 | 1 | 1 | 1 | 1 | 1 |  | 0 | 0 | 0 | 0 | 0 | 0 |
| Oribacterium sp. NK2B42 | 1 | 1 | 1 | 1 | 1 | 1 |  | 0 | 0 | 0 | 0 | 0 | 0 |
| Oribacterium sp. P6A1 | 1 | 1 | 1 | 1 | 1 | 1 |  | 0 | 0 | 0 | 0 | 0 | 0 |
| Peptostreptococcaceae bacterium pGA-8 | 1 | 1 | 1 | 1 | 1 | 1 |  | 0 | 0 | 0 | 0 | 0 | 0 |
| Peptostreptococcaceae bacterium VA2 | 1 | 1 | 1 | 1 | 1 | 1 |  | 0 | 0 | 0 | 0 | 0 | 0 |
| Peptostreptococcus anaerobius C | 1 | 1 | 0 | 0 | 0 | 1 |  | 0 | 0 | 0 | 0 | 0 | 0 |
| Peptostreptococcus sp. D1 | 1 | 1 | 1 | 1 | 1 | 1 |  | 0 | 0 | 0 | 0 | 0 | 0 |
| Prevotella brevis P6B11 | 1 | 1 | 1 | 1 | 1 | 1 |  | 0 | 0 | 0 | 0 | 0 | 0 |
| Prevotella bryantii C21a | 1 | 1 | 1 | 1 | 1 | 1 |  | 0 | 0 | 0 | 0 | 0 | 0 |
| Prevotella ruminicola Ga6B6 | 1 | 1 | 1 | 1 | 1 | 1 |  | 0 | 0 | 0 | 0 | 0 | 0 |
| Prevotella sp. AGR2160 | 1 | 1 | 1 | 1 | 1 | 1 |  | 0 | 0 | 0 | 0 | 0 | 0 |
| Prevotella sp. FD3004 | 1 | 1 | 1 | 1 | 1 | 1 |  | 0 | 0 | 0 | 0 | 0 | 0 |
| Prevotella sp. HUN102 | 0 | 0 | 0 | 0 | 0 | 0 |  | 0 | 0 | 0 | 0 | 0 | 0 |
| Prevotella sp. KH1P2 | 1 | 1 | 1 | 1 | 1 | 1 |  | 0 | 0 | 0 | 0 | 0 | 0 |
| Prevotella sp. KHP1 | 1 | 1 | 1 | 1 | 1 | 1 |  | 0 | 0 | 0 | 0 | 0 | 0 |
| Prevotella sp. KHP7 | 1 | 1 | 1 | 1 | 1 | 1 |  | 0 | 0 | 0 | 0 | 0 | 0 |
| Prevotella sp. LC2012 | 1 | 1 | 1 | 1 | 1 | 1 |  | 0 | 0 | 0 | 0 | 0 | 0 |
| Prevotella sp. MA2016 | 1 | 1 | 1 | 1 | 1 | 1 |  | 0 | 0 | 0 | 0 | 0 | 0 |
| Prevotella sp. P6B1 | 1 | 1 | 1 | 1 | 1 | 1 |  | 0 | 0 | 0 | 0 | 0 | 0 |
| Prevotella sp. P6B4 | 1 | 1 | 1 | 1 | 1 | 1 |  | 0 | 0 | 0 | 0 | 0 | 0 |
| Prevotella sp. RM4 | 1 | 1 | 1 | 1 | 1 | 1 |  | 0 | 0 | 0 | 0 | 0 | 0 |
| Prevotellaceae bacterium HUN156 | 1 | 1 | 1 | 1 | 1 | 1 |  | 0 | 0 | 0 | 0 | 0 | 0 |
| Proteiniclasticum ruminis DSM 24773 | 2 | 1 | 3 | 3 | 2 | 1 |  | 0 | 0 | 0 | 0 | 0 | 0 |
| Pseudobutyrivibrio ruminis ACV-9 | 1 | 1 | 1 | 1 | 1 | 1 |  | 1 | 1 | 1 | 1 | 1 | 1 |
| Pseudobutyrivibrio ruminis AD2017 | 1 | 1 | 1 | 1 | 1 | 1 |  | 1 | 1 | 1 | 1 | 1 | 0 |
| Pseudobutyrivibrio ruminis CF1b | 1 | 1 | 1 | 1 | 1 | 1 |  | 1 | 1 | 1 | 1 | 1 | 0 |
| Pseudobutyrivibrio ruminis DSM 9787 | 1 | 1 | 1 | 1 | 1 | 1 |  | 1 | 1 | 1 | 1 | 1 | 1 |
| Pseudobutyrivibrio ruminis HUN009 | 1 | 1 | 1 | 1 | 1 | 1 |  | 1 | 1 | 1 | 1 | 1 | 0 |
| Pseudobutyrivibrio sp AR14 | 1 | 1 | 1 | 1 | 1 | 1 |  | 1 | 1 | 1 | 1 | 1 | 1 |
| Pseudobutyrivibrio sp. ACV-2 | 1 | 1 | 1 | 1 | 1 | 1 |  | 1 | 1 | 1 | 1 | 1 | 0 |
| Pseudobutyrivibrio sp. C4 | 1 | 1 | 1 | 1 | 1 | 1 |  | 1 | 1 | 1 | 1 | 1 | 1 |
| Pseudobutyrivibrio sp. LB2011 | 1 | 1 | 1 | 1 | 1 | 1 |  | 1 | 1 | 1 | 1 | 1 | 1 |
| Pseudobutyrivibrio sp. MD2005 | 1 | 1 | 1 | 1 | 1 | 1 |  | 1 | 1 | 1 | 1 | 1 | 1 |
| Pseudobutyrivibrio sp. NOR37 | 1 | 1 | 1 | 1 | 1 | 1 |  | 1 | 1 | 1 | 1 | 1 | 1 |
| Pseudobutyrivibrio sp. OR37 | 1 | 1 | 1 | 1 | 1 | 1 |  | 1 | 1 | 1 | 1 | 1 | 1 |
| Pseudobutyrivibrio sp. UC1225 | 1 | 1 | 1 | 1 | 1 | 1 |  | 1 | 1 | 1 | 1 | 1 | 1 |
| Pseudobutyrivibrio sp. YE44 | 1 | 1 | 1 | 1 | 1 | 1 |  | 1 | 1 | 1 | 1 | 1 | 0 |
| Pseudobutyrivibrio xylanivorans DSM 10317 | 1 | 1 | 1 | 0 | 1 | 1 |  | 1 | 1 | 1 | 1 | 1 | 0 |
| Ruminobacter amylophilus DSM 1361 | 1 | 1 | 1 | 1 | 1 | 1 |  | 0 | 0 | 0 | 0 | 0 | 0 |
| Ruminobacter sp. RM87 | 1 | 1 | 1 | 1 | 1 | 1 |  | 0 | 0 | 0 | 0 | 0 | 0 |
| Ruminococcaceae bacterium AB4001 | 0 | 0 | 1 | 0 | 0 | 0 |  | 0 | 0 | 0 | 0 | 0 | 0 |
| Ruminococcaceae bacterium AE2021 | 0 | 0 | 1 | 0 | 0 | 0 |  | 0 | 0 | 0 | 0 | 0 | 0 |
| Ruminococcaceae bacterium D5 | 1 | 1 | 2 | 2 | 2 | 1 |  | 0 | 0 | 0 | 0 | 0 | 0 |
| Ruminococcaceae bacterium FB2012 | 1 | 1 | 1 | 1 | 1 | 1 |  | 0 | 0 | 0 | 0 | 0 | 0 |
| Ruminococcaceae bacterium KHP2 | 0 | 0 | 1 | 0 | 0 | 0 |  | 0 | 0 | 0 | 0 | 0 | 0 |
| Ruminococcaceae bacterium P7 | 1 | 1 | 1 | 1 | 1 | 1 |  | 0 | 0 | 0 | 0 | 0 | 0 |
| Ruminococcaceae bacterium YAD3003 | 0 | 0 | 1 | 1 | 0 | 0 |  | 0 | 0 | 0 | 0 | 0 | 0 |
| Ruminococcaceae bacterium YRB3002 | 0 | 0 | 1 | 1 | 0 | 0 |  | 0 | 0 | 0 | 0 | 0 | 0 |
| Ruminococcus albus AD2013 | 0 | 0 | 1 | 1 | 0 | 0 |  | 0 | 0 | 0 | 0 | 0 | 0 |
| Ruminococcus albus AR67 | 0 | 0 | 1 | 1 | 0 | 0 |  | 0 | 0 | 0 | 0 | 0 | 0 |
| Ruminococcus bromii YE282 | 1 | 1 | 1 | 1 | 1 | 1 |  | 0 | 0 | 0 | 0 | 0 | 0 |
| Ruminococcus flavefaciens AE3010 | 2 | 1 | 1 | 1 | 2 | 1 |  | 0 | 0 | 0 | 0 | 0 | 0 |
| Ruminococcus flavefaciens MA2007 | 2 | 1 | 1 | 1 | 2 | 1 |  | 0 | 0 | 0 | 0 | 0 | 0 |
| Ruminococcus flavefaciens MC2020 | 2 | 1 | 1 | 1 | 2 | 1 |  | 0 | 0 | 0 | 0 | 0 | 0 |
| Ruminococcus flavefaciens ND2009 | 2 | 1 | 1 | 1 | 2 | 1 |  | 0 | 0 | 0 | 0 | 0 | 0 |
| Ruminococcus flavefaciens SAb67 | 2 | 1 | 1 | 1 | 2 | 1 |  | 0 | 0 | 0 | 0 | 0 | 0 |
| Ruminococcus flavefaciens XPD3002 | 2 | 1 | 1 | 1 | 2 | 1 |  | 0 | 0 | 0 | 0 | 0 | 0 |
| Ruminococcus flavefaciens YAD2003 | 2 | 1 | 1 | 1 | 2 | 1 |  | 0 | 0 | 0 | 0 | 0 | 0 |
| Ruminococcus flavefaciens YL228 | 2 | 1 | 1 | 1 | 2 | 1 |  | 0 | 0 | 0 | 0 | 0 | 0 |
| Ruminococcus flavefaciens YRD2003 | 2 | 1 | 1 | 1 | 2 | 1 |  | 0 | 0 | 0 | 0 | 0 | 0 |
| Ruminococcus gnavus AGR2154 | 1 | 1 | 1 | 1 | 1 | 1 |  | 0 | 0 | 0 | 0 | 0 | 0 |
| Ruminococcus sp. FC2018 | 1 | 1 | 1 | 1 | 1 | 1 |  | 0 | 0 | 0 | 0 | 0 | 0 |
| Ruminococcus sp. HUN007 | 1 | 1 | 1 | 1 | 1 | 1 |  | 0 | 0 | 0 | 0 | 0 | 0 |
| Ruminococcus sp. NK3A76 | 1 | 1 | 1 | 1 | 1 | 1 |  | 0 | 0 | 0 | 0 | 0 | 0 |
| Ruminococcus sp. YE71 | 1 | 1 | 1 | 1 | 1 | 1 |  | 0 | 0 | 0 | 0 | 0 | 0 |
| Ruminococcus sp. YE78 | 1 | 1 | 1 | 1 | 1 | 1 |  | 0 | 0 | 0 | 0 | 0 | 0 |
| Sarcina sp. DSM 11001 | 1 | 1 | 1 | 1 | 1 | 1 |  | 0 | 0 | 0 | 0 | 0 | 0 |
| Selenomonas bovis 8-14-1 | 1 | 1 | 1 | 1 | 1 | 1 |  | 0 | 0 | 0 | 0 | 0 | 0 |
| Selenomonas ruminantium AB3002 | 1 | 1 | 1 | 1 | 1 | 1 |  | 0 | 0 | 0 | 0 | 0 | 0 |
| Selenomonas ruminantium AC2024 | 1 | 1 | 1 | 1 | 1 | 1 |  | 0 | 0 | 0 | 0 | 0 | 0 |
| Selenomonas ruminantium ATCC 12561 | 1 | 1 | 1 | 1 | 1 | 1 |  | 0 | 0 | 0 | 0 | 0 | 0 |
| Selenomonas ruminantium C3 | 1 | 1 | 1 | 1 | 1 | 1 |  | 0 | 0 | 0 | 0 | 0 | 0 |
| Selenomonas ruminantium GACV-9 | 1 | 1 | 1 | 1 | 1 | 1 |  | 0 | 0 | 0 | 0 | 0 | 0 |
| Selenomonas ruminantium lactilytica DSM 2872 | 1 | 1 | 1 | 1 | 1 | 1 |  | 0 | 0 | 0 | 0 | 0 | 0 |
| Selenomonas sp. AE3005 | 1 | 1 | 1 | 1 | 1 | 1 |  | 0 | 0 | 0 | 0 | 0 | 0 |
| Selenomonas sp. FC4001 | 1 | 1 | 1 | 1 | 1 | 1 |  | 0 | 0 | 0 | 0 | 0 | 0 |
| Selenomonas sp. ND2010 | 1 | 1 | 1 | 1 | 1 | 1 |  | 0 | 0 | 0 | 0 | 0 | 0 |
| Sharpea azabuensis DSM 18934 | 1 | 0 | 1 | 1 | 1 | 1 |  | 0 | 0 | 0 | 0 | 0 | 0 |
| Sharpea azabuensis DSM 20406 | 1 | 0 | 1 | 1 | 1 | 1 |  | 0 | 0 | 0 | 0 | 0 | 0 |
| Sharpea azabuensis KH1P5 | 1 | 0 | 1 | 1 | 1 | 1 |  | 0 | 0 | 0 | 0 | 0 | 0 |
| Sharpea azabuensis KH2P10 | 1 | 0 | 1 | 1 | 1 | 1 |  | 0 | 0 | 0 | 0 | 0 | 0 |
| Succinivibrio dextrinosolvens ACV-10 | 1 | 1 | 1 | 1 | 1 | 1 |  | 0 | 0 | 0 | 0 | 0 | 0 |
| Succinivibrio dextrinosolvens H5 | 1 | 1 | 1 | 1 | 1 | 1 |  | 0 | 0 | 0 | 0 | 0 | 0 |
| Treponema bryantii NK4A124 | 2 | 1 | 2 | 2 | 2 | 1 |  | 0 | 0 | 0 | 0 | 0 | 0 |
| Treponema bryantii XBD1002 | 2 | 1 | 2 | 2 | 2 | 1 |  | 0 | 0 | 0 | 0 | 0 | 0 |
| Treponema sp. C6A8 | 2 | 1 | 2 | 2 | 1 | 1 |  | 0 | 0 | 0 | 0 | 0 | 0 |

^1^Genomes not shown (*n* = 61) have no predicted subunits.
