# Supplementary material for: Electron transport phosphorylation in rumen butyrivibrios: unprecedented ATP yield for glucose fermentation to butyrate
Source: Front Microbiol. 2015 Jun 24;6:622. doi: 10.3389/fmicb.2015.00622 (PMC4478896; doi:10.3389/fmicb.2015.00622)
Supplement: Supplementary file 2 [file Image_1.PDF]

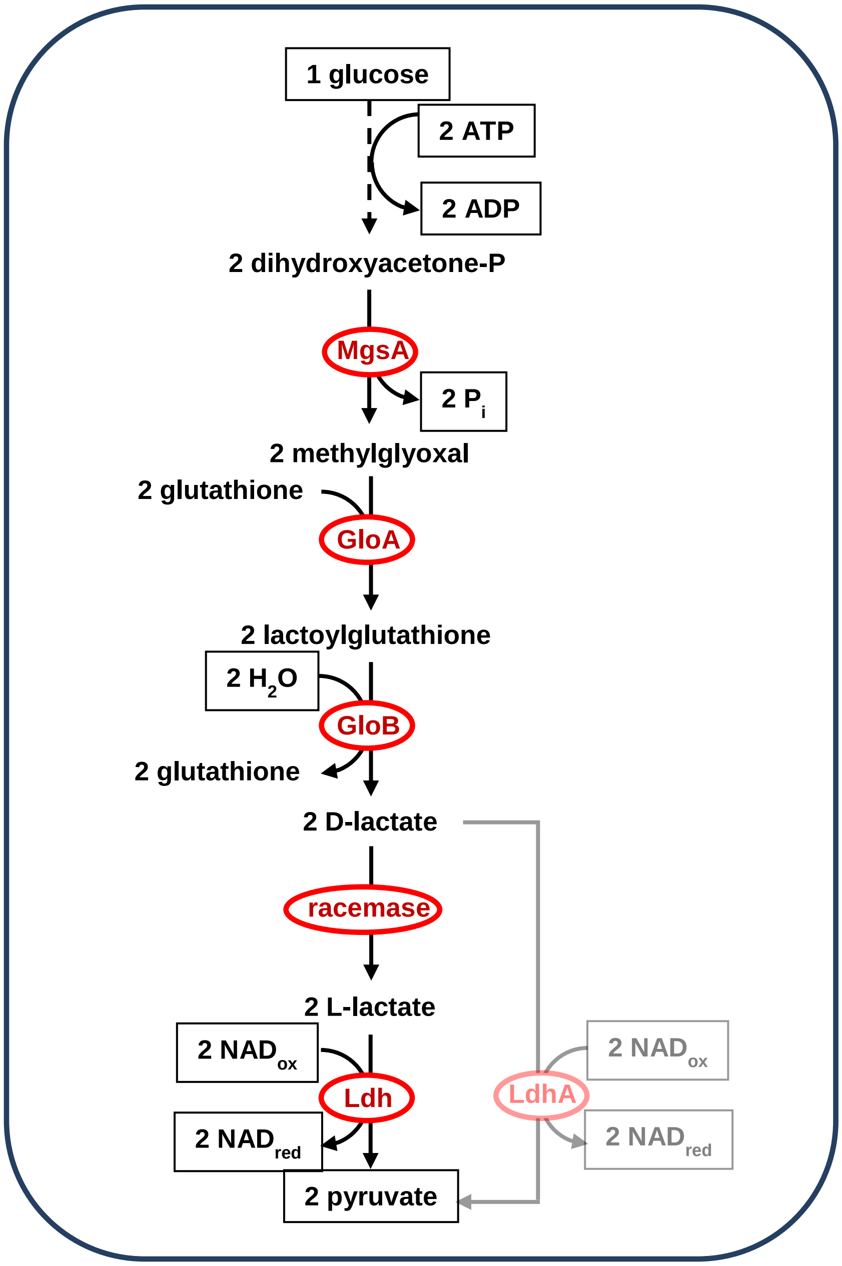

Net reaction:

$$1\ \text{glucose} + 2\ \text{ATP} + 2\ H_2O + 2\ NAD_{ox} =$$
$$2\ \text{pyruvate} + 2\ \text{ADP} + 2\ P_i + 2\ NAD_{red}$$

Supplementary Figure 1. Partial EMP pathway with methylglyoxal pathway in rumen butyrivibrios. The step with LdhA has been rendered partially transparent because it appears absent in most genomes. Steps with racemase and Ldh are depicted and could serve as an alternative to the step with LdhA. Yield is 4 ATP/glucose less than full EMP pathway. Dashed lines represent steps of the pathway condensed for brevity. See text for details and additional abbreviations.
