# Supplementary material for: Electron transport phosphorylation in rumen butyrivibrios: unprecedented ATP yield for glucose fermentation to butyrate
Source: Front Microbiol. 2015 Jun 24;6:622. doi: 10.3389/fmicb.2015.00622 (PMC4478896; doi:10.3389/fmicb.2015.00622)
Supplement: Supplementary file 3 [file Image_2.PDF]

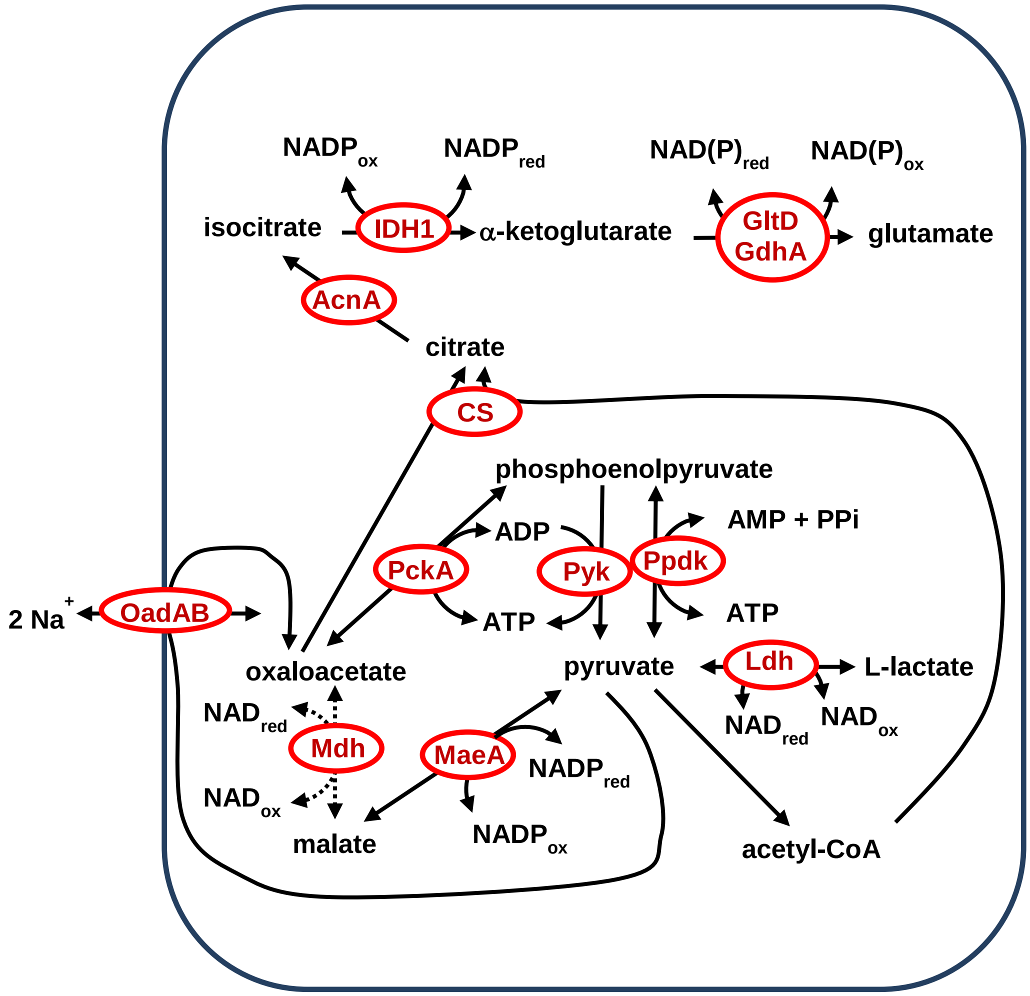

Supplementary Figure 2. A metabolic pathway for transhydrogenase activity. These reactions are based on a scheme for *Clostridium thermocellum* (Burton and Martin, 2012) and confirmed for potential activity in the butyrivibrios except for malate dehydrogenase (Mdh), which was lacking in all draft genomes (depicted with a dashed arrow). Oxaloacetate decarboxylase is expected to pump Na^+ out in the direction of pyruvate production (Repizo et al., 2013). See text for details and additional abbreviations.

Burton, E., and Martin, V.J. (2012). Proteomic analysis of *Clostridium thermocellum* ATCC 27405 reveals the upregulation of an alternative transhydrogenase-malate pathway and nitrogen assimilation in cells grown on cellulose. *Can J Microbiol* 58**,** 1378-1388.

Repizo, G.D., Blancato, V.S., Mortera, P., Lolkema, J.S., and Magni, C. (2013). Biochemical and genetic characterization of the *Enterococcus faecalis* oxaloacetate decarboxylase complex. *Appl Environ Microbiol* 79**,** 2882-2890.
